# Cyclin E-induced replicative stress drives p53-dependent whole-genome duplication

**DOI:** 10.1016/j.cell.2022.12.036

**Published:** 2023-01-20

**Authors:** Jingkun Zeng, Stephanie A. Hills, Eiko Ozono, John F.X. Diffley

**Affiliations:** 1 Chromosome Replication Laboratory, The Francis Crick Institute, London NW1 1AT, UK

## Abstract

Whole-genome duplication (WGD) is a frequent event in cancer evolution and an important driver of aneuploidy. The role of the p53 tumor suppressor in WGD has been enigmatic: p53 can block the proliferation of tetraploid cells, acting as a barrier to WGD, but can also promote mitotic bypass, a key step in WGD via endoreduplication. In wild-type (WT) p53 tumors, WGD is frequently associated with activation of the E2F pathway, especially amplification of *CCNE1*, encoding cyclin E1. Here, we show that elevated cyclin E1 expression causes replicative stress, which activates ATR- and Chk1-dependent G2 phase arrest. p53, via its downstream target p21, together with Wee1, then inhibits mitotic cyclin-dependent kinase activity sufficiently to activate APC/C^Cdh1^ and promote mitotic bypass. Cyclin E expression suppresses p53-dependent senescence after mitotic bypass, allowing cells to complete endoreduplication. Our results indicate that p53 can contribute to cancer evolution through the promotion of WGD.

## Graphical abstract

**Graphical abstract:**
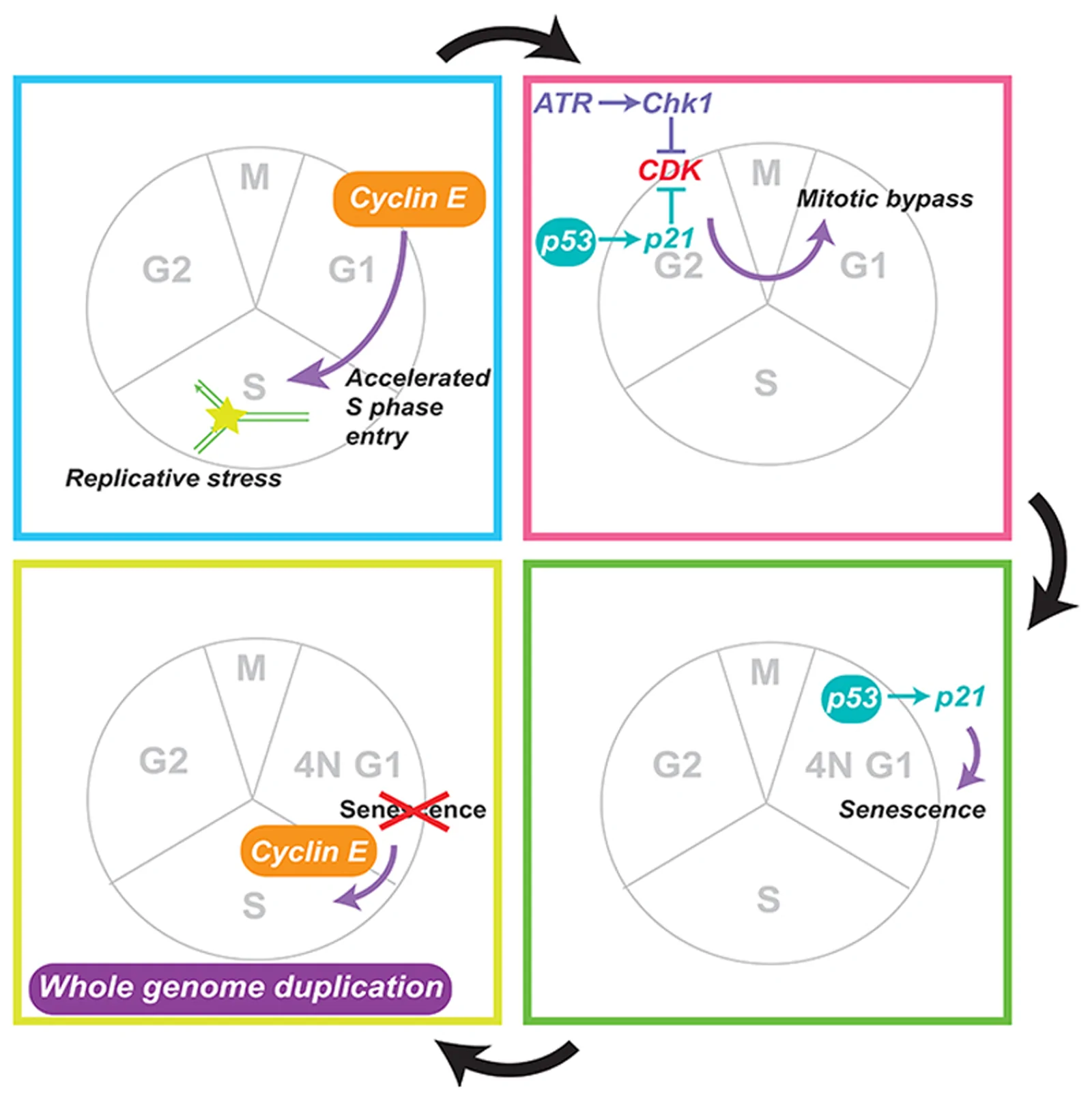

## Introduction

Almost 90% of human cancers exhibit aneuploidy.^1^ In many cancers, small numbers of chromosomes are gained or lost as a result of the mis-segregation of individual chromosomes in mitosis.^2^ However, in many other cancers, chromosome numbers are much higher, and their karyotypes are often described as being hypertriploid or sub-tetraploid.^2^ Such extensive aneuploidies are likely generated from a tetraploid intermediate.^2^ Approximately 30%–40% of human tumors have undergone whole-genome duplication (WGD) during their history, making it one of the most common single genomic events in oncogenesis,^3–5^ and WGD is generally associated with poor prognosis.^2,4^ Thus, understanding the causes and consequences of WGD is important for cancer biology.

WGD can occur by a variety of mechanisms. Cell-cell fusion can be induced by viruses including human papilloma virus (HPV), the causative agent of most cervical cancers.^6^ Failing to complete or exit mitosis (mitotic slippage)^7^ or defects in cytokinesis can also lead to WGD.^8^ Telomere attrition or persistent double-strand DNA breaks can promote WGD by mitotic bypass and endoreduplication.^9^ The relative importance of each of these pathways in different cancers is still largely unknown.

The tumor suppressor p53 protects cells from WGD by preventing the cell-cycle progression of G1 cells with a 4N DNA content. How this “tetraploid checkpoint” works is unknown,^10^ but it can be activated by mitotic slippage, cytokinesis blockage, and endoreduplication; moreover, WGD via endoreduplication after telomere attrition occurs only in cells lacking p53.^9^ One might, therefore, expect the loss of p53 to be essential for WGD to occur; however, large-scale genomics studies have shown that approximately half of the WGD events in cancer happen with wild-type (WT) p53 background.^4^ Also, p53 has been shown to promote mitotic bypass after genotoxic or oncogene stress.^11,12^ Although this generates tetraploid cells, these cells are senescent and therefore do not proliferate. How WGD happens in p53 proficient cells is still unclear. Deregulation of the E2F pathway is relatively common in p53-proficient tumors with WGD (~32%), especially amplification of the gene encoding cyclin E1 (CCNE1), suggesting a causal connection.^4^

Replicative stress is an early event in oncogenesis, resulting in the activation of DNA damage checkpoints after the acquisition of early cancer driver mutations.^13,14^ Among these drivers, deregulation of the E2F pathway, for example, by cyclin E expression, has been shown to induce replicative stress.^14–18^ The relationship between oncogene-induced replicative stress and WGD has not been explored. In this paper, we show how cyclin E can promote WGD in p53-proficient cells and how p53 can contribute to the generation of WGD.

## Results

### Cyclin E expression induces WGD by mitotic bypass in U2OS cells

**Figure 1:**
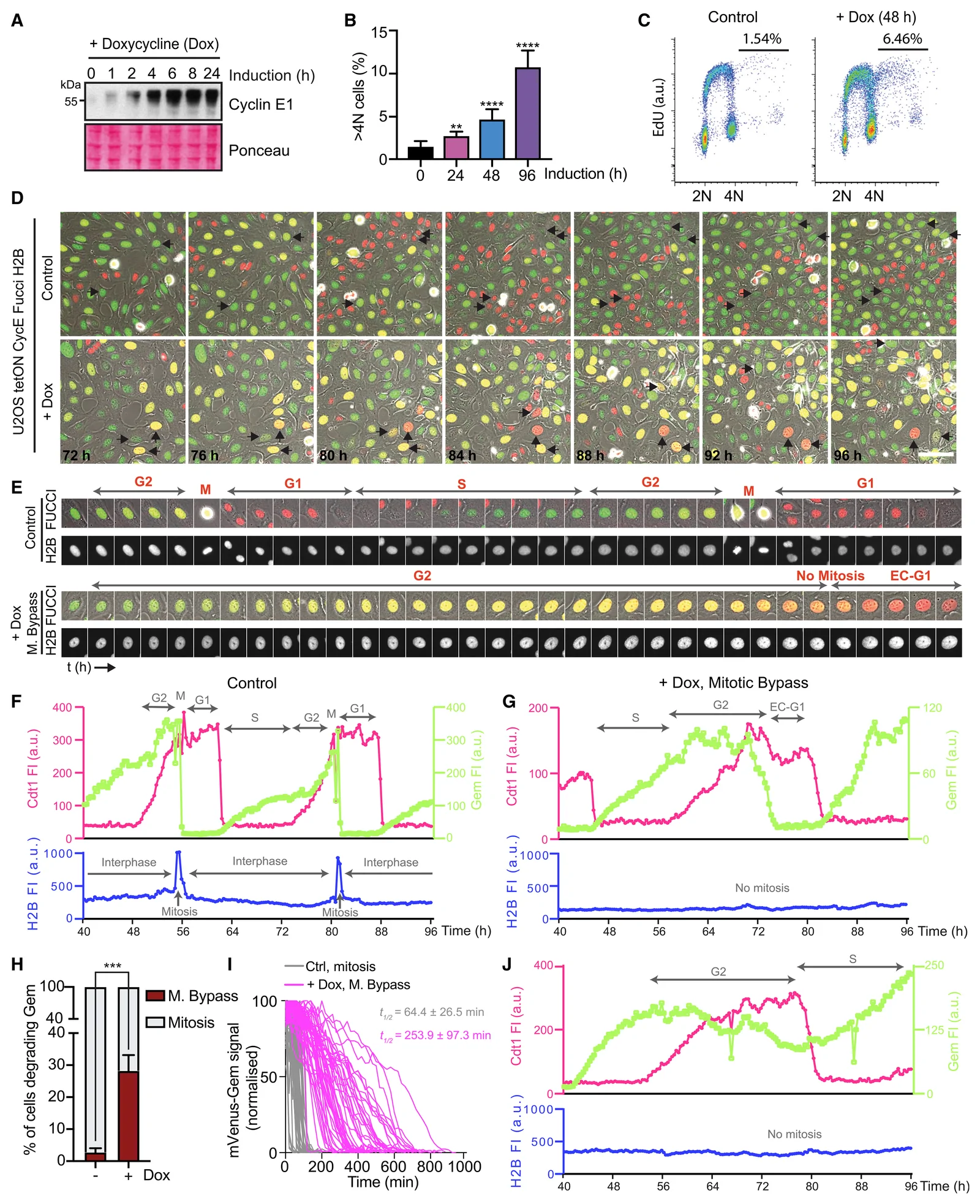
Cyclin E expression induces endoreduplication in U2OS cells (A) Immunoblots showing cyclin E1 (CycE) expression by doxycycline (Dox) treatment in U2OS tetON CycE cell line. (B) Quantification of cells with >4N DNA content following cyclin E induction over 96 h in U2OS, measured in % of total cells. Mean and standard deviations (SD) are shown (n = 3). Statistical significance: **p < 0.005 and ****p < 0.0001, unpaired t test. (C) FACS analysis of U2OS cells incorporating EdU after cyclin E induction. (D and E) Time-lapse imaging of U2OS cells expressing cyclin E. FUCCI and phase contrast images were merged. Selected still images are shown in (D) (see also Video S1). Black arrows of the same direction indicate individual tracked cells and their daughter cells. Tracking of example cells is shown in (E). Scale bars, 100 mm. (F and G) Temporal profiles of fluorescence intensities (FI) of mCherry-cdt1 and mVenus-Gem of cells. a.u., arbitrary unit. (H) Quantification of mitotic bypass, measured in % of cells that degraded geminin (% of cells completing S/G2). Mean and SDs were shown (n = 3), with >400 cells for each condition analyzed. Statistical significance: ***p < 0.001, unpaired t test. (I) Normalized time-course degradation of mVenus-Gem. Each line represents a single-cell tracking (n > 50 for each condition). Mean half-life (*t*_*1/2*_) with SDs are shown in the figure and main text. (J) Temporal profiles of a cyclin E-expressing U2OS cell that degraded mCherry-Cdt1 while maintaining high levels of mVenus-Gem.

To study the consequences of elevated cyclin E levels, we established a U2OS cell line expressing doxycycline-inducible

(“tetON”) full-length cyclin E1 (Figure 1A). U2OS is an osteosarcoma-derived cell line commonly used in DNA damage studies. Cyclin E expression in this cell line was somewhat faster than in a previously described “tetOFF” U2OS cell line expressing a cyclin E truncation (Figure S1A),^14^ but both cell lines express cyclin E to comparable levels (Figure S1A), which has previously been shown to be similar to levels seen in breast cancer cell lines containing increased copies of *CCNE1*.^14^ It was previously shown that a proportion of cells became >4N when expressing cyclin E,^14^ which was ascribed to partial genome re-replication. We confirmed this observation (Figures 1B and S1B), but by examining EdU incorporation (Figure 1C), we found that discrete G1, S, and G2 phases could be delineated at and above the 4N DNA content, apparently leading to complete replication. Therefore, these cells are undergoing a full cell cycle starting from a 4N DNA content, indicating that cyclin E expression can induce WGD. As an alternative approach to increasing cyclin E levels, we knocked down the FBXW7 (F box and WD repeat domain-containing 7) tumor suppressor that is required for cyclin E degradation and is frequently lost in cancer.^19^ We found that depletion of FBXW7 led to an increase in cyclin E expression, as well as to a small increase in c-Myc but not c-Jun or JunB expression (Figures S1C and S1D). Over a slightly longer time frame, FBXW7-depleted cells also underwent WGD (Figure S1E), indicating that even a moderate increase in cyclin E expression can also induce WGD.

To determine how WGD was generated following cyclin E expression, we used fluorescent ubiquitination-based cell-cycle indicator (FUCCI) live-cell imaging.^20^ In cells expressing fluorescently tagged, truncated versions of Cdt1 and geminin,^21^ one can distinguish G1 (high mCherry-Cdt1), S (high mVenus-Gem), and G2 (high mCherry-Cdt1 and high mVenus-Gem) phases of the cell cycle (Figure S1F). We also introduced the mTurquoise-H2B protein, which generates robust fluorescence throughout the cell cycle; this allows us to identify mitotic cells and enables better automated single-cell tracking. Control cells not expressing cyclin E entered and exited mitosis, as evidenced by the spike of mTurquoise-H2B fluorescence from chromosome condensation and by the rapid degradation of mVenus-Gem (Figures 1D–1F; Video S1). We saw no evidence of cell fusion, mitotic slippage, or cytokinesis defects in cyclin E-expressing cells. However, in roughly one-third of cyclin E-expressing cells, mVenus-Gem degradation occurred without mitosis (Figures 1D, 1E, 1G, and 1H; Video S1). In these cells, there was no evidence of chromosome condensation (mTurquoise-H2B spike) and no evidence of nuclear envelope breakdown (dispersal or leakage of the FUCCI markers from the nucleus), indicating that they transited directly from G2 to G1 phase. We call this G1 phase after mitotic bypass endoreduplication cycle G1 (EC-G1) (Figure 1G) to distinguish it from normal G1 phase. After a period of EC-G1, most cells then entered S phase, as seen by the degradation of mCherry-Cdt1 (Figure 1G). Taken together, these results indicate that cyclin E expression induces WGD via mitotic bypass and endoreduplication.

In contrast to the rapid mVenus-Gem degradation in mitosis of control cells (Figures 1F and 1I), mVenus-Gem degradation was much slower during mitotic bypass (Figures 1G and 1I): the halftime (*t*_1/2_) of degradation was 253.9 ± 97.3 min (median = 232.5 min) during mitotic bypass, roughly 4 times longer than control mitosis (64.4 ± 26.5 min mean *t*_*1/2*_, 71.6 min median *t*_*1/2*_). We found that there was also great variability in the length of time it took to degrade mVenus-Gem, ranging from ~2 to ~8 h (Figure 1I). Additionally, we saw many aberrant events, for example, where mVenus-Gem degradation either did not begin (Figure S1G) or began but did not go to completion (Figure 1J) before cells entered S phase (mCherry-Cdt1 degradation) (Figures 1J, S1G, and S1H). Thus, in contrast to normal mitosis, mitotic bypass is highly variable in length and often aberrant.

In mitotic cell cycles, two activators of the anaphase-promoting complex/cyclosome (APC/C)—Cdc20 and Cdh1—act sequentially in mitosis and G1, respectively,^22^ to degrade important cell-cycle substrates including geminin and cyclin B. In mitotic cell-cycle progression, Cdc20 is essential while Cdh1 plays a relatively minor role, but in endoreduplication cell cycles, including those generated by double-strand breaks (DSBs) or eroded telomeres, Cdh1 is crucial.^9^ Consistent with this, we found that depleting Cdh1 significantly reduced WGD in cyclin E-expressing cells (Figure S1I).

### The DNA damage checkpoint is required for WGD

**Figure 2:**
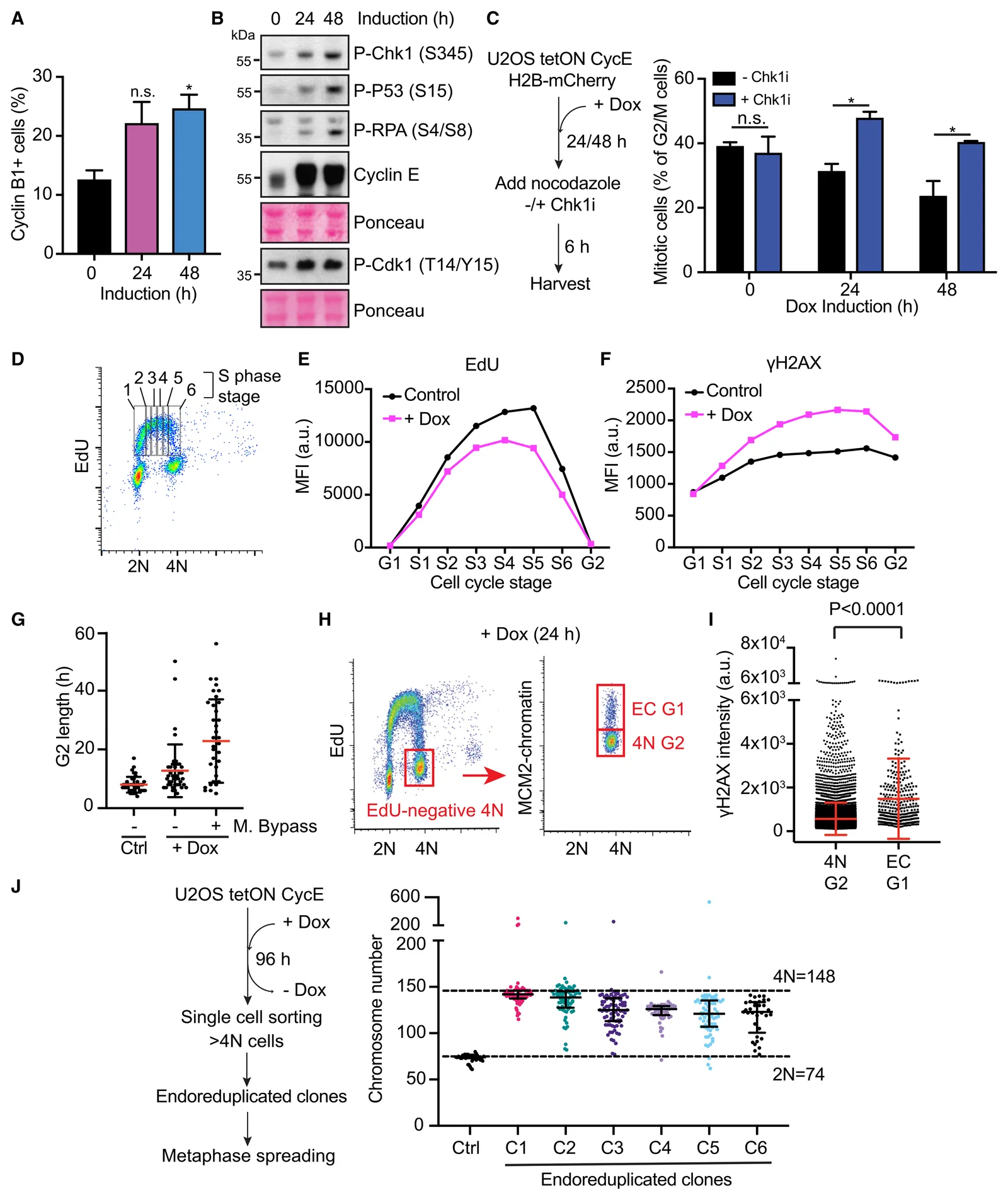
Replicative stress in cyclin E-expressing cells (A) Quantification of U2OS cells positive for cyclin B1 following cyclin E induction by FACS analysis. Mean and range are shown (n = 2). Statistical significance (*p < 0.05; n.s., non-significant) was examined by unpaired t test. (B) Immunoblots showing DNA damage markers after cyclin E induction in U2OS cells. (C) Left: schematic of the experimental approach. Mean percentages of mitotic cells with range are shown (n = 2). Statistical significance: *p < 0.05, unpaired t test. (D–F) Replicative stress in cyclin E-expressing cells. S phase (EdU+) was divided into 6 stages as shown in (D). The mean fluorescence intensities (MFI) of EdU and γH2AX were measured for each cell-cycle stage as shown in (E) and (F), respectively. a.u., arbitrary unit. (G) G2 lengths of individual cells from a representative FUCCI experiment in Figure 1D. (H and I) After 24-h cyclin E induction, EC-G1 and 4N G2 populations were identified by FACS analysis as illustrated in (H). EdU-negative cells at 4N DNA content were assumed to have finished replication and should be either in G2 or EC-G1. EC-G1 cells were then identified as those having loaded MCM. gH2AX level for individual cells in a representative experiment is shown in (I). Statistical significance: unpaired t test. (J) Karyotypes of endoreduplicated clones.

Cyclin E expression caused cells to accumulate in G2 phase over time, with an increase in cyclin B-positive cells (a marker of G2) (Figures 2A and S2A). In these cells, we saw increases in DNA damage markers (phospho-Chk1, phospho-p53, and phospho-RPA), increased inhibitory phosphorylation of the mitotic cyclin-dependent kinases 1 (CDK1) (Figure 2B), and a reduction in the proportion of G2 cells entering mitosis (Figure 2C black bars).

This is all consistent with previous work showing that cyclin E expression causes replicative stress.^14–18,23,24^ Cyclin E expression accelerates passage through G1 phase, causing cells to enter S phase prematurely (Figures S2B–S2D), before completion of the origin licensing program (Figures 2D, S2E, and S2F; Ekholm-Reed et al., 2004;^16^ Matson et al., 2017; ^18^ Tanaka and Diffley, 2002^25^)—this, in turn, reduces the rate of EdU incorporation during S phase (Figures 2D and 2E), prevents clearance of the minichromosome maintenance complex (MCM) from intragenic deposition sites^17^ and induces replication stress (Figures 2D and 2F), making S phase longer (Figure S2G). Similarly, depletion of FBXW7 reduces the rate of DNA synthesis and increases Chk1 phosphorylation (Figures S2H and S2I). The addition of a Chk1 inhibitor AZD7762 forced cyclin E-overexpressing G2-arrested cells into mitosis (Figure 2C) and increased the frequency of cells exhibiting aberrant mitosis (Figure S2J), indicating that the G2 arrest is dependent upon Chk1.

The mean length of G2 phase was 8 ± 3 h (median = 8 h) in control U2OS cells not expressing cyclin E, determined from live-cell imaging. G2 length was longer (13 ± 9 h; median = 10 h) in the subset of cyclin E-expressing cells that subsequently entered mitosis (Figure 2G). We did not observe EdU incorporation in these cells when they entered mitosis (phospho-histone H3 positive; Figure S2K), suggesting that DNA replication was completed before mitotic entry. Mean G2 length was even longer (23 ± 14 h; median = 21 h) in the subset of cells that subsequently bypassed mitosis and entered EC-G1 (Figure 2G), and these cells also bore higher levels of DNA damage markers (Figures 2H and 2I). Taken together, these results show that cyclin E expression induces replicative stress; cells with less stress can complete replication and enter mitosis after a transient G2 arrest. However, cells with more replicative stress remain arrested in G2 for extended periods; these cells ultimately undergo mitotic bypass, enter EC-G1 and then enter S phase to complete endoreduplication.

To characterize the outcome of endoreduplication, cells with >4N DNA content after cyclin E expression were separated by cell sorting, and individual clones were isolated, grown, and analyzed. Figure 2J shows that these clones had chromosome numbers between 74 (the diploid karyotype of U2OS cells) and 148 (the predicted chromosome number for U2OS cells after WGD) consistent with endoreduplication followed by chromosome loss. The chromosome number in individual cells from the clones shown in Figure 2L exhibited extensive variation compared with the control. This large variation in individual cell karyotypes suggests that changes in chromosome number (primarily chromosome loss) continued to occur during the growth of the clone. We also analyzed the first cell cycles after mitotic bypass by isolating cells with >4N DNA content after cyclin E expression by cell sorting. In these cells, the lengths of G2 and S phases were considerably longer (20 and 18 h, respectively) than in control cells (11 and 8 h) (Figures S2L and S2M). During the first mitosis following endoreduplication, 90% of the mitotic cells had bipolar spindles; these cells exhibited high levels of micronuclei, fragmented nuclei, and failed cytokinesis (Figures S2L, S2N, and S2O). Among the mitotic cells, 10% had multipolar spindles consistent with the presence of supernumerary centrosomes (Figures S2L, S2P, and S2Q),^26,27^ which likely also contributes to chromosome instability. These results suggest that in addition to progressive chromosome loss during colony growth, the first cell cycle after mitotic bypass is especially chaotic. The DNA content of EC-G1 cells was slightly less than G2 cells (Figures S2R and S2S), suggesting that replication was incomplete before mitotic bypass. Thus, some of the replicative stress may arise from the second round of replication occurring on an incompletely replicated genome. Regardless, these results are consistent with a recent publication showing that cells undergo high rates of replicative stress and DNA damage in the first S phase after induction of tetraploidy.^28^

### Replicative stress is a general inducer of endoreduplication in U2OS cells

**Figure 3:**
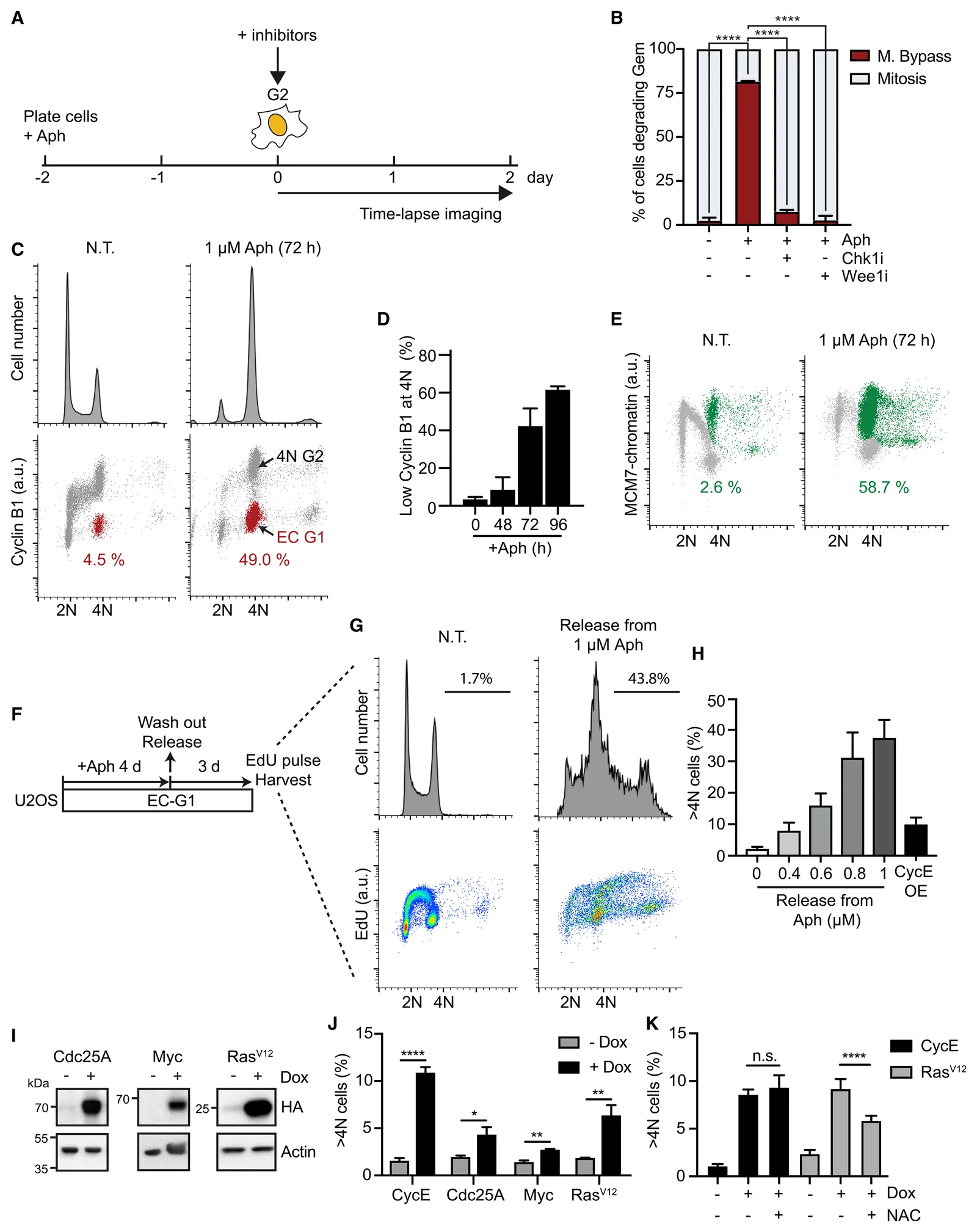
Mitotic bypass in aphidicolin-treated and oncogene-expressing cells (A and B) Schematic of the experiment approach is shown in (A). Quantification of U2OS cells that bypassed mitosis, measured in % of cells that degraded geminin (% of cells completing S/G2), is shown in (B) (see also Video S2). Mean and range are shown (n = 2, >200 cells for each condition analyzed). Statistical significance:****p <0.0001, Tukey’s method. (C–E) (C and E) U2OS cells treated with 1 µ M aphidicolin (Aph) for 72 h were analyzed for DNA content, cyclin B1 level, and chromatin-bound MCM7 level. EC-G1 cells were identified and labeled in red (C). Mean percentage of EC-G1 cells with range is shown in (D) (n = 2). Cells with >4N DNA content or cells with high levels of MCM loading at 4N are labeled in green (E). (F–H) Schematic of the experiment approach is shown in (F). Released cells were analyzed for DNA content and DNA synthesis (EdU) (G). Quantification of >4N cells incorporating EdU is shown with SDs in (H) (n = 3). (I) Immunoblots showing Cdc25A, Myc, or Ras^V12^ induction by Dox treatment in U2OS tetON cell lines. (J) Quantification of U2OS cells with >4N DNA content by FACS analysis after 96-h induction (Dox) of cyclin E (CycE), Cdc25A, Myc, or Ras^V12^. Mean percentages with SDs (n = 3) are shown. Statistical significance: *p < 0.05, **p < 0.005, and ****p < 0.0001, unpaired t test. (K) U2OS cells induced to express CycE or Ras^V12^ (+Dox) were incubated with 5mM N-acetyl cysteine (NAC). Quantification of cells with >4N DNA content by FACS analysis with SDs (n = 4) is shown. Statistical significance: ****p < 0.0001, Tukey’s method.

Results presented so far show that replicative stress caused by cyclin E expression induces WGD. To determine whether other forms of replicative stress could also induce endoreduplication or whether cyclin E expression plays any role in this process, in addition to generating replicative stress, we treated cells with the DNA polymerase inhibitor aphidicolin. Figures 3A and 3B and Video S2 show that this treatment is highly effective in inducing mitotic bypass in U2OS cells, even without cyclin E expression; nearly 80% of cells entered EC-G1 without mitosis after 72 h, as judged by mVenus-Gem degradation (Figure 3B; Video S2). This was accompanied by the accumulation of 4N cells with low cyclin B levels (Figures 3C and 3D) and re-loaded MCM (Figure 3E). mVenus-Gem degradation during aphidicolin-induced mitotic bypass was very slow with an average *t*_1/2_ of 229.8 ± 110 min (median = 211 min) (Figure S3A), similar to that seen with cyclin E expression (Figure S1D). Therefore, the slow G2 to EC-G1 transition seen in Figure 1G does not require continuous cyclin E expression. We saw an increase in DNA damage markers and an upregulation of p21 (Figure S3B), consistent with the generation of replicative stress. As with cyclin E expression, aphidicolin-induced bypass of mitosis required a Chk1-dependent G2 checkpoint since treatment with Chk1 or Wee1 inhibitors greatly reduced mitotic bypass and increased aberrant mitosis and micronucleus generation (Figures 3A, 3B, and S3C; Video S2). Like mitotic bypass after cyclin E expression, mitotic bypass after aphidicolin treatment also required Cdh1 (Figures S3D–S3F). When aphidicolin was removed after 96 h, >40% of the cells underwent endoreduplication (Figures 3F–3H). Thus, replicative stress induced by aphidicolin is sufficient to induce WGD in U2OS cells.

To explore the role of replicative stress in WGD further, we next tested whether replicative stress induced by other oncogenes could also induce WGD. To this end, we established U2OS cell lines (Figure 3I) in which expression of Cdc25A, Myc, or oncogenic Ras (Ras^V12^) can be induced. Figure 3J shows that all oncogenes tested generated elevated levels of WGD. Oncogenic Ras has been shown to induce replicative stress via the generation of reactive oxygen species.^29,30^ Consistent with this, the anti-oxidant N-acetyl cysteine (NAC) reduced DNA damage in Ras-expressing cells but not in cyclin E-expressing cells (Figure 3G). NAC also reduced WGD in Ras-expressing cells, but not cyclin E-expressing cells, consistent with the idea that it is Ras^V12^-induced replicative stress that drives WGD (Figure 3K). In addition, unlike cyclin E expression that reduced origin licensing (Figures 2D, S2E, and S2F), oncogenic Ras did not affect MCM loading (Figure 3H). Taken together, these results show that replicative stress acts as a general driver of WGD, with different forms of replicative stress, including oncogene-induced replicative stress, capable of inducing endoreduplication.

### Cyclin E drives WGD in hTERT-RPE1 cells

**Figure 4:**
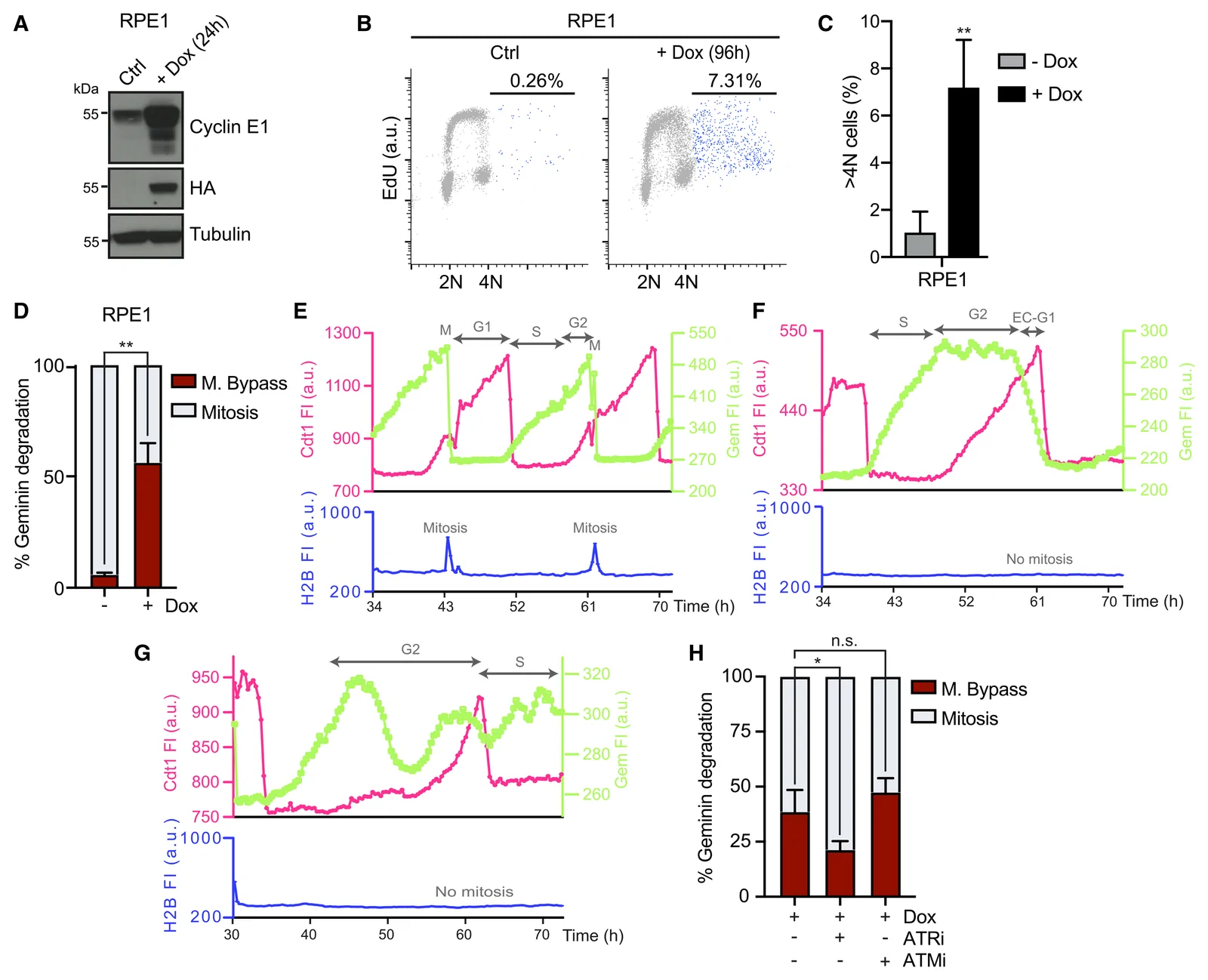
Cyclin E expression induces endoreduplication in RPE1 cells (A) Immunoblots showing cyclin E induction (+Dox) in RPE1 tetON CycE cells. (B) FACS analysis of RPE1 cells incorporating EdU after 96-h cyclin E expression. (C) Quantification of cells with >4N DNA content, measured in % of total cells, in (B) with SDs (n = 4). Statistical significance: **p < 0.005, unpaired t test. (D) Quantification of cyclin E-expressing RPE1 cells (+Dox) that bypassed mitosis, measured in % of cells that degraded geminin (% of cells completing S/G2), with SDs is shown (n = 3, >150 cells for each condition analyzed). (E and F) Temporal profiles of the fluorescence intensities (FI) of mCherry-cdt1 and mVenus-Gem of a control RPE1 cell and a Dox-treated RPE1 cell bypassing mitosis. a.u., arbitrary unit. Statistical significance: **p < 0.005, unpaired t test. (G) Temporal profiles of an example Dox-treated RPE1 cell degrading mCherry-cdt1 with high levels of mVenus-Gem. (H) Schematic of the experiment approach is shown in Figure 3A, except that the initial treatment was Dox instead of Aph. Mean percentages of RPE1 cells that bypassed mitosis with SDs are shown (n = 3, >200 cells analyzed for each condition). Statistical significance: *p < 0.05, unpaired t test.

U2OS cells are transformed and have likely already undergone a WGD event as evident from their hypertriploid karyotype (see Figure 2J). Moreover, although U2OS cells express WT p53, their actual p53 status is complicated at least in part due to the absence of *CDKN2A* expression,^31^ which encodes the alternative reading frame (ARF) Mdm2 inhibitor in addition to the p16 CDK inhibitor.^32^ We therefore wanted to determine whether cyclin E could induce WGD in a more normal, diploid cell line. hTERT-RPE1 (hereafter RPE1) cells are untransformed, neardiploid, retinal epithelial cells with WT p53 that have been immortalized by telomerase expression. We established an RPE1 cell line expressing doxycycline-inducible (tetON) full-length cyclin E1 (Figure 4A), analogous to the U2OS cell line described above. We found that cyclin E-expressing RPE1 cells underwent WGD with similar kinetics to the U2OS cell line (Figures 4B and 4C). These RPE1 cells expressing cyclin E1 had reduced origin licensing, reduced rate of DNA synthesis, and increased DNA damage markers (Figures S4A–S4C) similar to U2OS cells (Figures 2B, 2D, 2E, S2F, and S2G), indicative of replicative stress and G2 checkpoint activation.

Control RPE1 cells not expressing cyclin E entered and exited mitosis, whereas roughly half of cyclin E-expressing RPE1 cells bypassed mitosis (Figures 4D–4F). mVenus-Gem degradation during mitotic bypass in cyclin E-expressing RPE1 cells was also very slow with a *t*_1/2_ of 261.1 ± 134.1 min (mean ± SD, median = 231.8 min) (Figure S4D). We also saw many aberrant events, where mVenus-Gem degradation began but did not go to completion before cells entered S phase (mCherry-Cdt1 degradation) (Figures 4G and S4E). The addition of an ATR inhibitor but not an ATM inhibitor significantly reduced mitotic bypass, indicating that mitotic bypass is primarily dependent upon ATR (Figures S4F and 4H). Together, these experiments show that expression of cyclin E can induce WGD via mitotic bypass in an untransformed, p53-proficient, diploid cell line.

### p53 is required for mitotic bypass

**Figure 5:**
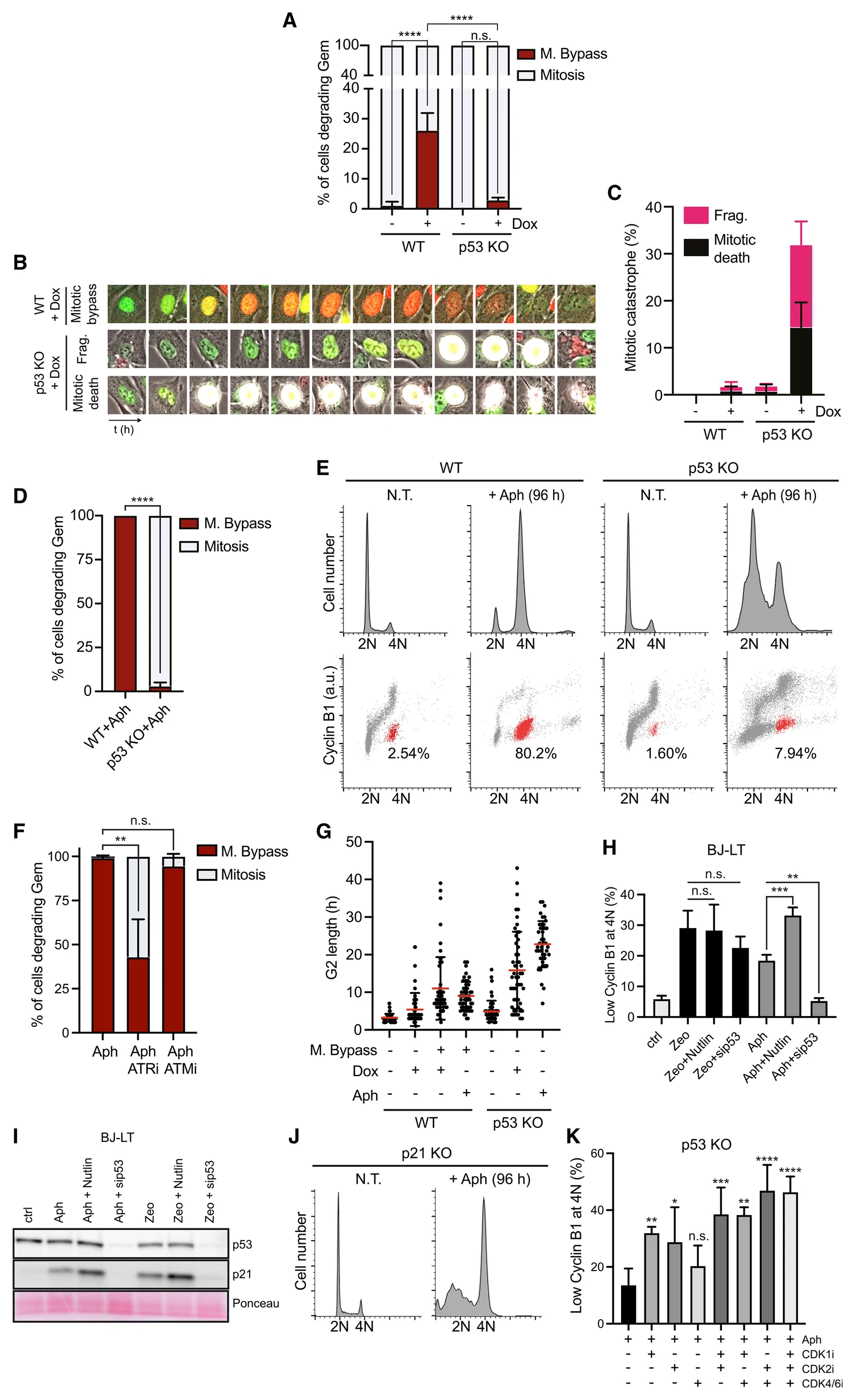
p53 knockout abolishes mitotic bypass in aphidicolin-treated and cyclin E-expressing RPE1 cells (A–C) RPE1 WT and p53 KO cells were induced to express cyclin E (+ Dox) and imaged for 96 h. Quantification of cells that bypassed mitosis with SDs is shown in (A) (n = 3). Selected images of example cells are shown in (B) (see also Videos S3 and S4). Quantification of nuclear fragmentation (Frag.) and mitotic death is shown with SDs in (C). At least 300 cells for each condition were analyzed. Statistical significance: ****p < 0.0001, method. (D) RPE1 WT and p53 KO cells were treated with 1 µM aphidicolin (Aph) and imaged for 72 h (see also Video S5). Quantification of mitotic bypass with SDs is shown (n = 3, >200 cells for each condition analyzed). Statistical significance: ****p < 0.0001, unpaired t test. (E) RPE1 WT and p53 KO cells treated with 1 µM Aph were analyzed by FACS at 96 h. EC-G1 cells were identified and labeled in red. (F) Schematic of the experiment approach is shown in Figure 3A. Quantification of RPE1 cells that bypassed mitosis with SDs is shown (n = 3, >200 cells for each condition analyzed). Statistical significance: **p < 0.005, Tukey’s method. (G) Measured G2 length of cells in (A) and (D) from single representative experiments. (H and I) BJ-LT cells treated with 0.5 µM aphidicolin (Aph) or 50 mg/ml zeocin (Zeo) for 72 h were supplemented with 2 µM Nutlin (at time 24 h) or p53 siRNA (at time 0) before being analyzed by immunoblots and FACS. Immunoblots showing p53 and p21 expression are shown in (I). Mean percentages of cells with low cyclin B1 at 4N DNA content with SDs are shown in (H) (n = 3). Statistical significance: **p < 0.005 and ***p < 0.001, Tukey’s method. (J) RPE1 p21 KO cells treated with 1 µM Aph were analyzed by FACS for DNA content. (K) RPE1 p53KO cells treated with 1 µM Aph for 48 h were supplemented with inhibitors of CDK1, CDK2, or CDK4/6 for a further 24 h before FACS analysis. Mean percentages of cells with low cyclin B1 at 4N DNA content with SDs are shown (n = 3). Statistical significance: *p < 0.05, **p < 0.005, ***p < 0.001, and ****p < 0.0001, Tukey’s method.

The results in the previous section show that cyclin E expression can drive WGD in p53-proficient RPE1 cells. To test whether this is also true in p53-deficient cells, we inactivated the TP53 gene in the RPE1 cyclin E-expressing cell line using CRISPR-Cas9 (Figure S5A). We found similar cyclin E expression levels, DNA damage marker upregulation, and reduction in the rate of DNA synthesis in p53-positive and p53-knockout RPE1 cells, following doxycycline induction (Figures S5B and S5C). Whereas approximately 25% of cyclin E-expressing p53-positive cells by-passed mitosis, mitotic bypass and WGD were almost completely suppressed in the p53-knockout RPE1 cells (Figures 5A and S5D; Videos S3 and S4). Instead of bypassing mitosis, a high proportion of these p53-knockout cells entered into catastrophic mitosis (Figures 5B and 5C). Therefore, the presence of p53 is not only permissive for mitotic bypass and endoreduplication following replicative stress, but it is in fact essential for the process.

Similar to U2OS cells, RPE1 cells treated with aphidicolin efficiently bypassed mitosis (mVenus-Gem degradation) and accumulated in EC-G1 (high mCherry-Cdt1), with 4N DNA content and low cyclin B levels (Figures 5D, 5E, and S5E; Video S5). Therefore, replicative stress is sufficient to cause mitotic bypass in p53-proficient cells. Similar to cyclin E expression, the addition of an ATR inhibitor but not an ATM inhibitor significantly reduced mitotic bypass in aphidicolin (Figure 5F). In RPE1 cells lacking p53, aphidicolin treatment induced similar levels of DNA damage markers as in p53-proficient cells (Figure S5F) but did not result in mitotic bypass (Figures 5D and S5E). Instead, there was an increase in cells with <2N DNA content; cells entered catastrophic mitosis resulting in nuclear fragmentation and death (Figures 5E, S5G, and S5H; Video S5). Taken together, these results show that replicative stress-driven mitotic bypass, whether caused by cyclin E or aphidicolin, requires p53.

This requirement is not restricted to RPE1 cells: p53-positive HCT116 cells accumulated in EC-G1 (4N DNA content with low cyclin B) (Figures S5I and S5J), while cells lacking p53 including p53-knockout U2OS, p53-depleted HCT116, and HeLa cells, which are p53 deficient, exhibited either prolonged G2 arrest with high cyclin B levels (p53-knockout U2OS) or an increase in cells with <2N DNA content and nuclear fragmentation (p53-depleted HCT116 cells and HeLa cells) (Figures S5K–S5Q). We also found that aphidicolin-induced mitotic bypass occurred in a non-transformed fibroblast cell line IMR90 and that this mitotic bypass was greatly reduced after the knockdown of p53 (Figure S5R).

One could explain the aberrant mitotic entry described above if p53 contributed to the G2 checkpoint arrest after replicative stress.^33^ However, we found by live-cell imaging that both cyclin E-expressing cells and aphidicolin-treated cells remained in G2 phase much longer in the p53-knockout cells before they entered aberrant mitosis than in the p53-positive cells before they bypassed mitosis, indicating that the G2 checkpoint was still activated and maintained in the absence of p53 (Figure 5G). Therefore, rather than being involved in maintaining the G2 arrest, our results indicate that p53 is actively required for replicative stress-driven mitotic bypass.

A requirement for p53 in WGD appears at odds with previous work showing that mitotic bypass and endoreduplication after telomere attrition or double-strand DNA breaks (DSB-driven mitotic bypass) occurred in p53-deficient cells. For their work, Davoli et al. used p53^-/-^ MEFs and human cells in which p53 was inactivated by viral oncoproteins such as SV40 large T antigen (SV40LT).^9^ Indeed, we could confirm that zeocin induced mitotic bypass in BJ cells immortalized with SV40LT (Figure 5H). Moreover, the amount of mitotic bypass induced by zeocin in these cells was not enhanced by Nutlin, an Mdm2 inhibitor, and was not significantly decreased by p53 siRNA, indicating that zeocin-induced mitotic bypass does not require p53 (Figure 5H), consistent with the work of Davoli et al.^9^. Aphidicolin also induced mitotic bypass in these cells, but in sharp contrast to zeocin treatment, mitotic bypass induced by aphidicolin was markedly increased by Nutlin treatment and eliminated by p53 siRNA (Figure 5H). Despite the expression of SV40LT, it is clear these cells can still mount a p53 response since they exhibited an increase in p21 after zeocin or aphidicolin treatment, which was enhanced by Nutlin treatment and eliminated by p53 siRNA (Figure 5I). Thus, in the same cell line, DSB-driven mitotic bypass is independent of p53, whereas replicative stress-driven mitotic bypass absolutely requires p53. Zeocin or POT1 depletion also induced mitotic bypass or WGD in U2OS cells (Figures S6A– S6D), which likely has a dampened p53 response. By contrast, in p53-positive RPE1 cells, zeocin treatment or POT1 depletion primarily induced a G1 arrest (Figures S6E and S6F), consistent with DNA damage-induced G1 checkpoint activation. Nonetheless, there was also an increase in mitotic bypass as evidenced by the increase in cells with a 4N DNA content and low cyclin B (Figures S6E and S6F).

### p53 promotes mitotic bypass via p21 inhibition of CDK

p21, GADD45α, and 14-3-3 α are key downstream G2/M targets of p53,^34^ so we depleted each individually in RPE1 cells treated with aphidicolin. Only p21 depletion reduced the accumulation of cells in EC-G1 (4N DNA content, low cyclin B) as p53 depletion did (Figure S6G). We also generated p21-knockout RPE1 cells (Figure S6H) and found that they behaved very similarly to p53-knockout cells: instead of the accumulation of cells in EC-G1 (Figure S6I), there was an increase in cells with <2N DNA content in aphidicolin (Figure 5J). Knockout of p21 in U2OS cells or depletion of p21 in IMR90 cells and HCT116 also almost completely abolished the accumulation of EC-G1 cells (Figures S5K–S5O and S5R). Depletion of p21 in HCT116 cells treated with aphidicolin caused accumulation of <2N cells and increased nuclear fragmentation, consistent with cells being forced into aberrant mitoses (Figures S5P and S5Q). Therefore, p21 is the major mediator of p53’s function in mitotic bypass.

p21 is an inhibitor of CDK. We wondered whether chemical inhibition of CDK could bypass the requirement for p53 in inducing mitotic bypass. To test this, we added inhibitors of CDK1, CDK2, or CDK4/6 to G2-arrested p53-knockout RPE1 cells treated with aphidicolin. Figure 5K shows that inhibition of CDK1 or CDK2 significantly increased the accumulation of cells in EC-G1. Coincubation of two or three of the CDK inhibitors further increased EC-G1 accumulation (Figure 5K). Taken together, these results support the idea that mitotic bypass is initiated when CDK activities are inhibited in G2 by Wee1 and p21 to a sufficiently low level to allow activation of APC/C^Cdh1^.

### Cyclin E expression prevents and reverses senescence entry

**Figure 6:**
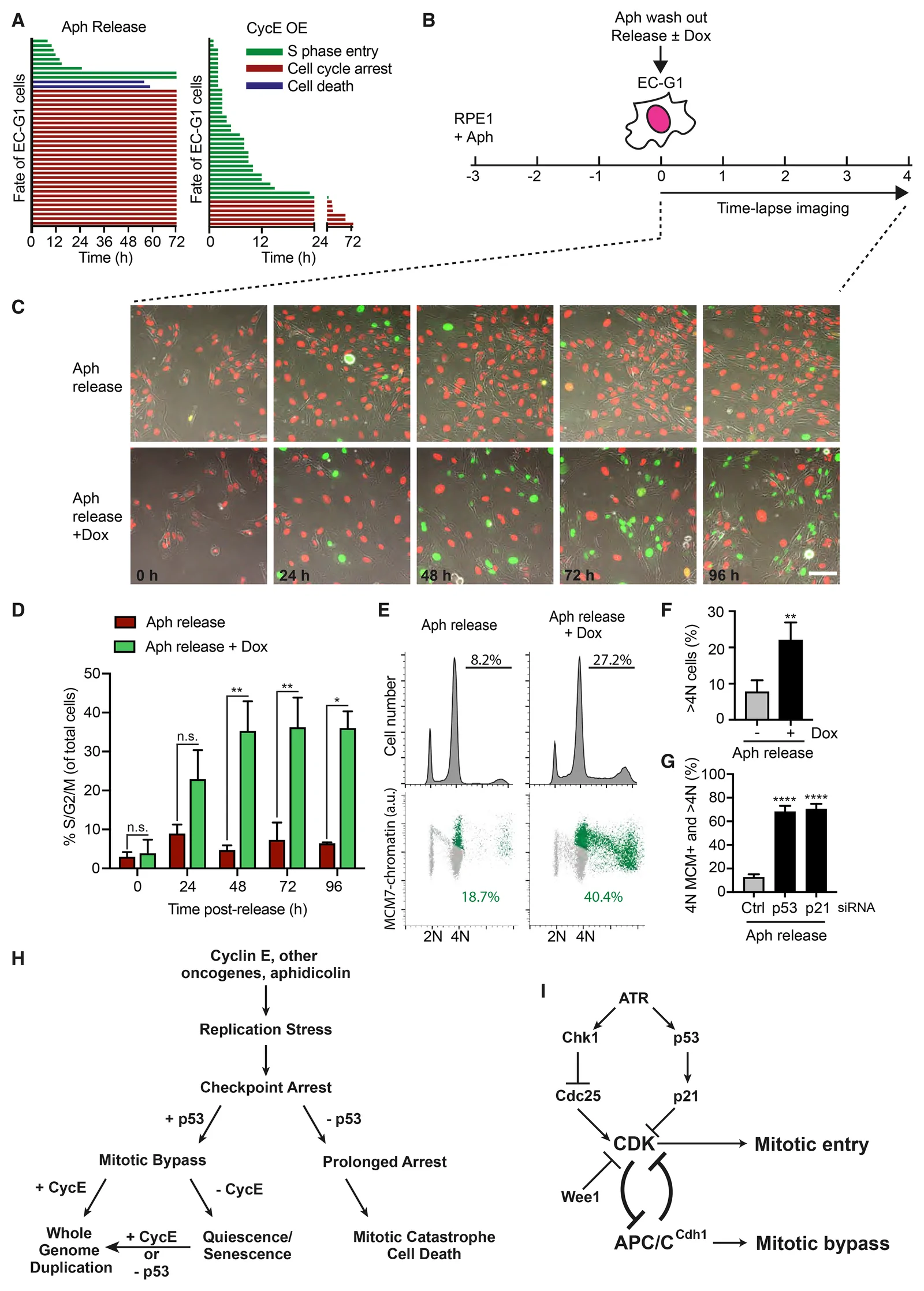
CycE expression re-establishes endoreduplication of senescent EC-G1 cells (A) Individual cell fates of RPE1 EC-G1 cells released from 0.5 µM aphidicolin (Aph) treatment or generated with CycE expression (represented by single-colored lines) are shown. (B–D) Schematic of the experiment approach is shown in (B). 0.5 µM Aph was used. Still images at indicated time points are shown in (C) (see also Video S6). Scale bars, 100 mm. The mean percentage with SEMs of cells in S/G2/M phase at indicated time points is shown in (D) (n = 3). Statistical significance: *p < 0.05 and **p < 0.005, Šidák’s method. (E and F) RPE1 cells were treated as in (B) and analyzed by FACS at 96 h post Aph release for DNA content and MCM loading. Cells with >4N DNA content or with high levels of MCM loading at 4N are labeled in green. Quantification of >4N cells incorporating EdU is shown with SDs in (F) (n = 4). Statistical significance: **p < 0.005, unpaired t test. (G) Cells were treated as in (B) and released with siRNAs for 96 h. Mean percentages with SDs of cells with a high MCM level at 4N DNA content or with >4N DNA content are shown (n = 3). Statistical significance: ****p < 0.0001, Tukey’s method. (H and I) Model of whole-genome duplication driven by oncogene-induced replicative stress in p53-positive cells. Details of the model are described in the text.

Aphidicolin-induced RPE1 EC-G1 cells did not reload MCM or proliferate after release from aphidicolin (Figures 6A and S7A), in contrast to U2OS (Figures 3F–3H) or RPE1 cells expressing cyclin E (Figure 4B). RPE1 EC-G1 cells generated by aphidicolin treatment were positive for β-galactosidase activity (Figure 7B), suggesting that they have become senescent. Previous work has shown that transient activation of p53 in G2 can cause mitotic bypass and promote entry into senescence.^11,12^ Since cyclin E expression, in contrast to aphidicolin treatment, induces WGD in p53-proficient RPE1 cells (Figure 6A), cyclin E must prevent this entry into senescence; but, can cyclin E expression drive cells that have already entered senescence back into the cycle? To test this, we asked whether the expression of cyclin E after mitotic bypass induced by aphidicolin could drive the senescent EC-G1 cells into the cell cycle. Figures 6B–6H show that a large fraction of these EC-G1 cells were induced to enter the cell cycle (Figures 6C and 6D; Video S6), re-license and rereplicate their genomes (Figure 6E), and accumulate as endore-duplicated cells (Figure 6F). Knocking down Rb in these EC-G1 cells was less efficient than cyclin E expression at driving them into cycle, but it did result in the re-licensing of DNA (Figure S7C). Senescent EC-G1 cells generated by aphidicolin treatment and then driven back into cell cycle by cyclin E expression, or cells with >4N DNA content after cyclin E expression alone, were separated by cell sorting, and individual clones could be isolated. All but one of these clones exhibited sub-tetraploid chromosome numbers and extensive chromosome number variation (Figure S7D), similar to that seen in U2OS endoreduplicated clones. One clone (C4) had clearly undergone an additional round of endoreplication and had a sub-octaploid chromosome number. These results indicate that the senescent EC-G1 state is not irreversible. This conclusion is reinforced by the fact that the knockdown of either p53 or p21 in EC-G1 cells also greatly induced cell-cycle re-entry and re-replication (Figures 6G and S7E). Taken together, these results indicate that p53-dependent mitotic bypass induced by replicative stress does not induce an irreversible arrest, but it rather induces a state with some hallmarks of senescence that can be reversed by alterations in the Rb or p53 pathways.

## Discussion

Our results, summarized in Figures 6H and 6I, describe a pathway for WGD via endoreduplication that requires p53. The pathway begins with the generation of replicative stress, a common consequence of oncogene expression. In the case of cyclin E expression, replicative stress arises from perturbation of the replication origin licensing system caused by the shortened G1 phase (Macheret and Halazonetis, 2018;^17^ Matson et al., 2017;^18^ and this study). However, Ras^V12^ does not reduce origin licensing (Figure S3H); instead, it generates replicative stress through the generation of reactive oxygen species.^29,30^ Our results show that both types of stress can induce mitotic bypass and WGD. Moreover, exogenous sources of replicative stress like aphidicolin can also induce mitotic bypass. Since many anti-cancer drugs work by interfering with DNA replication, we speculate that drug treatments may promote WGD, even in p53-proficient cells, which may have implications for cancer evolution after chemotherapy.

Replicative stress induces mitotic bypass after a prolonged checkpoint-dependent G2 arrest, with ATR being primarily responsible for the DNA damage signal. This mitotic bypass requires the activation of the G1 form of the APC/C, APC/C^Cdh1^, which is normally repressed by CDK activity. The DNA damage checkpoint in human cells blocks entry into mitosis via Wee1-dependent inhibition of mitotic CDK. This inhibition of CDK is sufficient to prevent mitotic entry and is essential for mitotic bypass, but it is not sufficient to activate APC/C^Cdh1^. Instead, mitotic bypass requires an additional CDK inhibitor, p21, whose accumulation is also dependent upon checkpoint activation, in this case, via p53 in a pathway parallel to Wee1. The time taken to transit from G2 (high mVenus-Gem) to G1 (low mVenus-Gem) varied widely in our experiments, suggesting that some or all of the feedback loops involved in the switch-like activation of the APC/C at the metaphase to anaphase transition in a normal cell cycle are not fully operational.^35^

The mitotic bypass seen after continued cyclin E expression has similar kinetics to the bypass induced by aphidicolin, indicating that oscillations in cyclin E-CDK2 are not essential for endoreduplication. This contrasts with naturally occurring endoreduplication cycles in *Drosophila*, which require a low cyclin E period to promote origin licensing and a high cyclin E period to drive replication.^36^ In human cells, it appears that cyclin E expression does not directly inhibit licensing. For example, overexpression of cyclin E in U2OS cells does not affect the rate of MCM loading during G1 phase (Figures S2B and S2D). Moreover, cyclin E plays a positive role in licensing by preventing APC/C-dependent degradation of Cdc6.^37^ The reduced MCM loading seen in these cells when they enter S phase is because cyclin E expression shortens G1 phase and therefore reduces the time available for licensing (Figures S2B and S2C). These results are also consistent with biochemical experiments showing that human cyclin A-CDK2 but not cyclin E-CDK2 phosphorylation can inhibit APC/C^Cdh1^ activity *in vitro*.^38^ Thus, our results are consistent with the idea that cyclin E overexpression directly inhibits neither replication origin licensing nor APC/C^Cdh1^ activity.

After mitotic bypass induced by aphidicolin, p53-proficient RPE1 cells arrest in a senescence-like state. This is very likely related to previous work showing that transient induction of p53 in G2 triggers entry into senescence after mitotic bypass.^11,12^ Cyclin E expression prevents this entry into senescence and can drive these senescent cells to complete endoreduplication. Cancers that have deregulated the E2F pathway, for example, by amplifying *CCNE1*, should, based on our findings, be primed to endoreduplicate without entering senescence. In other cases, where mitotic bypass and senescence occur before driver acquisition, subsequent E2F deregulation or p53 loss might drive cells back into cycle from senescence as tetraploid cells.

Previous work has shown that DSBs can drive WGD in cells lacking p53, whereas our results show that WGD driven by replicative stress requires p53. In both cases, the underlying mechanism of WGD is the same—extended checkpoint-dependent CDK inactivation allows APC/C^Cdh1^ activation and subsequent mitotic bypass. However, the DSB-driven mechanism requires p53 deficiency because DSBs cause p53-dependent G1-arrest, which prevents WGD; p53 is not required because DSBs generate a strong enough checkpoint signal to cause mitotic bypass without p21. The mechanism we describe here for replicative stress requires p53 proficiency because the additional CDK inhibition from p21 is essential for mitotic bypass; p53 loss is not required because replicative stress does not induce p53-dependent G1 arrest. The DSB-driven mechanism requires telomere attrition to generate the checkpoint signal and requires p53 inactivation; the mechanism we describe requires genetic alteration in the cyclin E pathway to generate the checkpoint signal and to prevent senescence. These genetic events are all common in cancer and thus both pathways may play important roles in cancer.

Our results show that viral oncogenes may not always fully inactivate p53. For example, BJ cells transformed by SV40 large T antigen can still express p21 when treated with zeocin or aphidicolin (Figure 5H). Also, both DSB-driven and replicative stress-driven mechanisms can work in cells like BJ-LT that have dampened p53 function. It will be interesting to assess the ability of common p53 mutants to promote both DSB-driven as well as replicative stress-driven endoreduplication. p53 is classically considered to be a tumor suppressor gene; however, p53 null mutants are rare in cancer, and studies on the distribution of p53 mutations in cancer have led to a more nuanced vision in which p53 mutants can also contribute to oncogenesis.^39^ Our results fit into this view of p53 and suggest that p53 may actually contribute to cancer evolution by promoting replicative stress-driven WGD.

### Limitations of the study

The relevance for tumorigenesis of the mechanism for WGD described in this study has not been directly addressed either in animal models or by human cancer genetics. This study primarily used acute cyclin E expression, which may be different from the gradual accumulation of *CCNE1* gene expression during amplification over several generations.

## Star★Methods

### Key Resources Table

**Table:** 

REAGENT or RESOURCE	SOURCE	IDENTIFIER
Antibodies		
Mouse monoclonal anti-Cyclin E1	Santa Cruz Biotechnology	Cat# sc-247, RRID:AB_627357
Mouse monoclonal anti-beta Actin	Santa Cruz Biotechnology	Cat# sc-81178, RRID:AB_2223230
Rabbit polyclonal anti-Phospho-p53 (Ser15)	Cell Signaling Technology	Cat# 9284, RRID:AB_331464
Rabbit monoclonal anti-Phospho-Chk1 (Ser345)	Cell Signaling Technology	Cat# 2348, RRID:AB_331212
Rabbit polyclonal anti-Phospho-RPA32 (S4/S8)	Bethyl	Cat# A300-245A, RRID:AB_210547
Mouse monoclonal anti-HA.11 Epitope Tag	BioLegend	Cat# 901533, RRID:AB_2801249
Rabbit polyclonal anti-HA Epitope Tag	Santa Cruz Biotechnology	Cat# sc-805, RRID:AB_631618
Mouse monoclonal anti-p53	Santa Cruz Biotechnology	Cat# sc-126, RRID:AB_628082
Mouse monoclonal anti-alpha-Tubulin	Sigma-Aldrich	Cat# T5168, RRID:AB_477579
Mouse monoclonal anti-MCM7	Santa Cruz Biotechnology	Cat# sc-56324, RRID:AB_1125697
Rabbit monoclonal anti-Cyclin B1	Abcam	Cat# ab32053, RRID:AB_731779
Mouse monoclonal anti-phospho-Histone H2A.X (Ser139)	Millipore	Cat# 05-636, RRID:AB_309864
Rabbit polyclonal anti-POT1	Novus Biologicals	Cat# NB500-176, RRID:AB_10000829
Rabbit polyclonal anti-FBXW7	Proteintech	Cat# 55290-1-AP RRID:AB_2881300
Anti-p-CDK1 T14/Y15	In-house by Julian Gannon	N/A
Anti-Cdh1	In-house by Julian Gannon	N/A
Goat polyclonal Anti-Mouse Immunoglobulins	Agilent	Cat# P0447, RRID:AB_2617137
Donkey polyclonal Anti-Rabbit IgG (H+L)	Jackson ImmunoResearch Labs	Cat# 711-035-152, RRID:AB_10015282
Goat polyclonal anti-Rabbit IgG (H+L) Cross- Adsorbed Secondary Antibody, Alexa Fluor 555	Thermo Fisher Scientific	Cat# A-21428, RRID:AB_2535849
Goat polyclonal anti-Mouse IgG (H+L) Highly Cross-Adsorbed Secondary Antibody, Alexa Fluor 555	Thermo Fisher Scientific	Cat# A-21424, RRID:AB_141780
Chemicals, peptides, and recombinant proteins		
Dulbecco’s Modified Eagle’s Medium (DMEM) - high glucose	Gibco	Cat# 41966052
Lipofectamine RNAiMAX	ThermoFisher	Cat# 13778150
Opti-MEM Reduced Serum Medium, GlutaMAX Supplement	ThermoFisher	Cat# 51985034
Aphidicolin	Sigma Aldrich	Cat# A0781
AZD 7762	Axon MEDCHEM	Cat# 1399
MK 1775	Axon MEDCHEM	Cat# 1494
KU-55933	Selleckchem	Cat# S1092
VE-822	Selleckchem	Cat# S7102
Abemaciclib	Selleckchem	Cat# S7158
RO-3306	Merck	Cat# SML0569
CVT-313	Cambridge Bioscience	Cat# B1137
Nocodazole	Sigma Aldrich	Cat# M1404
Colcemid	ThermoFisher	Cat# 15212012
Doxycycline	Sigma Aldrich	Cat# D9891
DyeCycle Ruby	ThermoFisher	Cat# V10309
Hoechst 33342	ThermoFisher	Cat# 62249
DAPI	Sigma Aldrich	Cat# D9542
Alexa Fluor 488 NHS Ester	ThermoFisher	Cat# A20000
Vectashield Antifade Mounting Medium with DAPI	Vector Laboratories	Cat# H-1200
Protease Inhibitor Cocktail	Sigma Aldrich	Cat# 11873580001
JetPRIME	Polyplus	Cat# 114-15
Lipofectamine 3000	ThermoFisher	Cat# L3000008
Zeocin	Invivogen	Cat# ant-zn-1
Blasticidin	Invivogen	Cat# ant-bl-05
Critical commercial assays		
QIAquick Gel Extract kit	QIAGEN	Cat# 28706
QIAprep Spin Miniprep Kit	QIAGEN	Cat# 27106
Click-iT® EdU Alexa Fluor® 647 Flow Cytometry Assay Kit	ThermoFisher	Cat# C10424
Experimental models: Cell lines		
Human: U2OS	ATCC	HTB-96
Human: hTERT RPE1	ATCC	CRL-4000
Human: HCT116	ATCC	CCL-247
Human: IMR90	ATCC	CCL-186
Human: U2OS TetON CycE	This paper	Available upon request
Human: U2OS TetON Ras^12V^	This paper	Available upon request
Human: U2OS TetON c-Myc	This paper	Available upon request
Human: U2OS TetON cdc25A	This paper	Available upon request
Human: U2OS TetON CycE p53KO	This paper	Available upon request
Human: U2OS TetON CycE p21KO	This paper	Available upon request
Human: U2OS TetON CycE Fucci H2B	This paper	Available upon request
Human: RPE1 TetON CycE	This paper	Available upon request
Human: RPE1 TetON CycE p53KO C1 & C2	This paper	Available upon request
Human: RPE1 TetON CycE p21KO	This paper	Available upon request
Human: RPE1 TetON CycE Fucci	This paper	Available upon request
Human: RPE1 TetON CycE Fucci H2B	This paper	Available upon request
Human: RPE1 TetON CycE p53KO Fucci	This paper	Available upon request
Human: U2OS CycE TetOFF	Laboratory of Jiri Bartek	N/A
Human: BJ-LT	Laboratory of Mariia Yuneva	N/A
Oligonucleotides		
SMARTpool On-TARGETplus FZR1 (Cdh1) siRNA	Dharmacon	L-015377-00
siGENOME TP53 siRNA	Dharmacon	D-003329-26
SMARTpool siGENOME CDKN1A siRNA (p21)	Dharmacon	M-003471-00
SMARTpool siGENOME RB1 siRNA	Dharmacon	M-003296-03
SMARTpool siGENOME GADD45A siRNA	Dharmacon	M-003893-02
SMARTpool siGENOME SFN siRNA (14-3-3sigma)	Dharmacon	M-005180-00
SMARTpool siGENOME FBXW7 siRNA	Dharmacon	M-004264-02
SMARTpool siGENOME POT1 siRNA	Dharmacon	M-004205-01
Control siRNA siGL2 against Firefly luciferase: CGU ACG CGG AAU ACU UCG AUU	Ohrt et al.^40^	N/A
gRNA for TP53 knockout targeting exon 4: CCATTGTTCAATATCGTCCG	This paper	N/A
gRNA for TP53 knockout targeting exon 5: TCCTCAGCATCTTATCCGAG	This paper	N/A
gRNA for CDKN1A knockout: CCATTAGCGCATCACAGTCG	This paper	N/A
Recombinant DNA		
Plasmid: Fucci(CA)2	Sakaue-Sawano et al.^21^	N/A
Plasmid: pCSII EF1a hH2B-Turq	Silvia Santos	N/A
Plasmid: psPax2	Addgene	12260
Plasmid: pMD2.G	Addgene	12259
Plasmid: pSpCas9(BB)-2A-Puro (PX459) V2.0	Addgene	62988
Plasmid: PX459-TP53-exon4	This paper	Available upon request
Plasmid: PX459-TP53-exon5	This paper	Available upon request
Plasmid: PX459-CDKN1A	This paper	Available upon request
Plasmid: pcDNA4/TO	Invitrogen	V102020
Plasmid: pcDNA4/TO-CycE	This paper	Available upon request
Plasmid: pcDNA4/TO-Ras	This paper	Available upon request
Plasmid: pcDNA4/TO-cMyc	This paper	Available upon request
Plasmid: pcDNA4/TO-cdc25A	This paper	Available upon request
Software and algorithms		
FlowJo 10.8	FlowJo, LLC	https://www.flowjo.com/
MATLAB	Mathworks	https://www.mathworks.com/
ImageJ 1.53	NIH	RRID:SCR_001935
FIJI	NIH	RRID: SCR_002285
TrackMate plugin for FIJI	Tinevez et al.^41^	https://github.com/fiji/TrackMate
Prism 8	GraphPad	https://www.graphpad.com/scientific-software/prism/
FUCCI imaging analysis FIJI macro	This paper	https://github.com/zeng-j-k/Cell-FUCCI-analysis.git

## Resource Availability

### Lead contact

Further information and requests for resources and reagents should be directed to and will be fulfilled by the lead contact, John F.X. Diffley (john.diffley@crick.ac.uk).

### Materials availability

All unique/stable reagents generated in this study will be made available on request to the lead contact but may require a completed Materials Transfer Agreement.

## Experimental Model and Subject Details

### Cell lines and culture conditions

All cells in this study were cultured using DMEM (Gibco, 41966052) supplemented with 10% fetal bovine serum in an ambient-controlled incubator at 37 °C and 5% CO_2_. No antibiotics were supplemented unless specified. U2OS, hTERT RPE1, HCT116 and Hela cells were used in this study. U2OS TetON CycE, U2OS TetON Ras^12V^, U2OS TetON c-Myc, U2OS TetON cdc25A, U2OS TetON CycE p53KO, U2OS TetON CycE p21KO, U2OS TetON CycE Fucci H2B, RPE1 TetON CycE, RPE1 TetON CycE p53KO (clone #1&2, clone 1 was used unless specified), RPE1 TetON CycE p21KO, RPE1 TetON CycE Fucci, RPE1 TetON CycE Fucci H2B and RPE1 TetON CycE p53KO Fucci were generated for this study. U2OS CycE TetOFF was previously described in^14^ and was kindly gifted from Thanos D. Halazonetis. See Plasmids and Cell Lines for details of construction of cell lines.

## Method Details

### Plasmids and cell lines

Stable TetON cell lines were constructed by random plasmid integration. Human coding sequences of Cyclin E1, c-Myc, H-Ras^V12^ and cdc25A were cloned in to the pcDNA4/TO vector (Invitrogen) respectively with a hemagglutinin (HA) tag at the N-terminus. T-REx-U2OS cells were obtained from Invitrogen. T-REx-RPE1 cells stably expressing the Tet repressor were constructed by transfecting hTERT RPE1 cells with the pcDNA6/TR plasmid (Invitrogen) and cells were selected in medium containing 5 mg/ml Blasticidin. For creation of TetON cells, T-REx-U2OS cells or T-REx-RPE1 cells were transfected with Lipofectamine 3000 (Invitrogen) or JetPRIME (Polyplus) using the pcDNA4/TO constructs carrying genes of interest. Transformed cells were selected in 200-500 mg/ml Zeocin and clones were tested for Doxycycline dependent gene expression.

For creation of *TP53* knockout cells, we used the following gRNA sequences: 5’ CCATTGTTCAATATCGTCCG 3’ targeting exon 4 and 5’ TCCTCAGCATCTTATCCGAG 3’ targeting exon 6, cloned into the pSpCas9(BB)-2A-Puro (PX459) V2.0 vector (Addgene, 62988). The resultant constructs were co-transfected into U2OS TetON CycE or RPE1 TetON CycE cells. Transfected cells were single cell sorted into 96-well plates four days post puromycin selection. Successful *TP53* knockout in single cell clones was verified by immunoblotting, PCR and sanger sequencing. For *CDKN1A* knockout, we used a gRNA sequence 5’ CCATTAGCGCATCACAGTCG 3’ and followed procedures described above.

The Fucci(CA)2 plasmid^21^ carrying a Cdt1 fragment, mCherry tagged, and a geminin fragment, mVenus tagged, was introduced into U2OS TetON CycE, RPE1 TetON CycE and RPE1 TetON CycE p53KO C1 by lentiviral transduction. Stable clonal transformants positive for fluorescent signals were obtained by single cell sorting into 96-well plates. The pCSII EF1a hH2B-Turq plasmid carrying mTurquoise-tagged human H2B, provided by Dr. Silvia Santos, was introduced into U2OS TetON CycE Fucci and RPE1 TetON CycE Fucci cells by lentiviral transduction.

### RNA Interference and small molecule inhibitors

Knockdown studies were performed using SMARTpool On-TARGETplus FZR1(Cdh1) siRNA (Dharmacon, L-015377-00), siGENOME TP53 siRNA (Dharmacon, D-003329-26), SMARTpool siGENOME CDKN1A siRNA (Dharmacon, M-003471-00), SMARTpool siGENOME GADD45A siRNA (Dharmacon, M-003893-02), SMARTpool siGENOME SFN siRNA (Dharmacon, M-005180-00), SMARTpool siGENOME FBXW7 siRNA (Dharmacon, M-004264-02), SMARTpool siGENOME POT1 siRNA (Dharmacon, M-004205-01), SMARTpool siGENOME RB1 siRNA (Dharmacon, M-003296-03) and a control siRNA siGL2 against Firefly luciferase^40^ with sequence CGU ACG CGG AAU ACU UCG AUU. siRNAs were transfected at 40 nM final concentration using Lipofectamine RNAiMAX (Invitrogen) and OptiMEM (Invitrogen). The following small molecule compounds were used: aphidicolin from *Nigrospora sphaerica* (Sigma Aldrich), AZD 7762 (CHK1 inhibitor, Axon MedChem, used at 50 nM working concentration), MK 1775 (WEE1 inhibitor, Axon MedChem, used at 1 μM working concentration), KU-55933 (ATM inhibitor, Selleckchem, used at 10 μM working concentration), VE-822 (ATR inhibitor, Selleckchem, used at 200 nM working concentration), Abemaciclib (CDK4/ 6 inhibitor, Selleckchem, used at 500 nM working concentration), RO-3306 (CDK1 inhibitor, Merck, used at 7 μM working concentration), CVT-313 (CDK2 inhibitor, Cambridge Bioscience, used at 10 mM working concentration), nocodazole (Sigma Aldrich), colcemid (Thermo), Doxycycline (Sigma Aldrich).

### Metaphase spreading

Cells were stained with Hoechst and FACS-sorted for DNA content. Single-cell clones were selected, and chromosome numbers were counted by metaphase spreading. Endoreduplicated clones were grown in medium supplemented with 167 ng/ml colcemid (Thermo) for 3 h. Cells were trypsinised, re-suspended in 75 mM KCl at 37 °C for 10 min with gentle vortexing, and fixed in Carnoy’s fixative (3:1 methanol:glacial acetic acid). Cells were washed two more times with Carnoy’s fixative before spreading on slides. Mitotic samples were mounted in a mounting medium containing DAPI (Vector Laboratories) to visualise chromosomes.

### Live-Cell imaging

Cells were grown on 4-well polymer bottom slides (80446, Ibidi) in DMEM (Gibco, 41966052) containing 10% FBS with 1% Pen/Strep. Sorted cells were allowed to settle for at least 5 h prior to imaging. Time-lapse live-cell imaging was performed using a Nikon Eclipse Ti inverted microscope equipped with a custom humidified enclosure (Okolabs) that maintains temperature at 37 °C and CO_2_ at 5%.

The Nikon Perfect Focus System (PFS) was used for autofocus. Phase-contrast and fluorescent images were taken every 20 min using ImageJ-μ Manager software with a 20x objective. Filter sets and exposure times were optimised so that no phototoxicity or photobleaching was observed in cells. Medium in wells was replenished every 2 or 3 days. Image processing was performed using FIJI software. Cell lines used are: U2OS tetON CycE Fucci H2B-mTurQ in Figures 1D and 3A; RPE1 TetON CycE Fucci H2B in Figure 4D; RPE1 TetON CycE Fucci in Figure 4H; RPE1 TetON CycE Fucci and RPE1 TetON CycE p53KO Fucci in Figure 5A; RPE1 TetON CycE Fucci and RPE1 TetON CycE p53KO Fucci in Figure 5D; RPE1 TetON CycE Fucci in Figure 5F; RPE1 TetON CycE Fucci in Figure 6B.

### Automated cell tracking

Image analyses for U2OS cells in Figures 1D–1J and RPE1 cells in Figures 4D–4G were analysed by an in-house script-based automated cell tracking pipeline. Acquired images were filtered and background subtracted in FIJI (1.53c) before tracking using a plugin, Trackmate.^41^ H2B-mTurquoise channel was used for tracking cell nuclei, and parameters were optimised for effective tracking of nuclei as following: the LoG detector was used with default parameters except using a radius of 11 µm for U2OS cells, and a radius of 9 µm for RPE1 cells. The Simple LAP Tracker was used with a max linking distance of 15, a max gap closing distance of 15 and a max frame gap of 2.

Fluorescence intensities were calculated on identified nuclear regions of H2B-mTurquiose, cdt1-mCherry and geminin-mVenus images. Cells showing red (mCherry+, mVenus-), green (mCherry-, mVenus+) and yellow (mCherry+, mVenus+) were assigned to G1, S and G2 phases respectively. Upon mitosis, one of the daughter cells is selected for tracking. MATLAB was used to plot fluorescence intensity changes over time for individual identified cells with tracks longer than 36 h. Mitosis was characterised as an abrupt increase in H2B-mTurquiose signal caused by condensation of chromosomes, in parallel with abrupt disappearance of mVenus-geminin signal. Mitotic bypass was characterised as disappearance of mVenus-geminin with no increase in H2B-mTurquiose signal. Image analyses for U2OS cells in Figures 3A and 3B and RPE1 cells in Figures 5A–5D and 5F were performed by manual tracking of cells.

### Numerical analysis

To estimate degradation rates of mVenus-Gem, FUCCI datasets were exported, and time courses were excised around local maxima and minima. Excised intensity data were normalised and scaled to 0-100, and fitted to the logistic growth equation below^21^:

$$Normalised\;Intensity\;=\frac{100}{1+e^{\left(-k\left(t-t_{1/2}\right)\right)}}$$

*k* is the rate of degradation of mVenus-Gem signal with a unit of 1/minute. *t*_*1/2*_ is half-life of mVenus-Gem degradation with a unit of minute, at which the signal reaches half of the maximum. Curves were fitted using lsqcurvefit function in MATLAB.

### Flow cytometry and cell sorting

Multiplexed flow cytometry analysis using fluorescent cell barcoding, combined with EdU, antibody and DNA staining, was performed as previously described.^42^ Up to 6 samples treated with different conditions were barcoded in each experiment to allow unbiased subsequent staining of the combined samples. For detection of S phase progression, cells pulsed with 10 mM EdU for 30 min were harvested and stained with Click-iT chemistry using Click-iT EdU Alexa Fluor 647 Flow Cytometry Assay Kit (Thermo, C10424) according to the manufacturer’s instructions. For DNA content analysis, cells were treated with 100 µg/mL RNase A and stained with 1 µg/mL DAPI. For MCM loading analysis, cell chromatin fractions were extracted using CSK buffer (10 mM HEPES-KOH pH 7.9, 100 mM NaCl, 3 mM MgCl_2_, 1 mM EGTA, 300 mM sucrose, 1% BSA, 0.2% Triton X-100, 1 mM DTT, 1X Roche Complete protease inhibitor cocktail) before fixation and staining. Data were analysed using FlowJo software. Cell doublets were excluded for all analyses. See Antibodies for details of epitope staining. EC-G1 cells are identified as cells having low cyclin B1 level at 4N DNA content.

Non-EC and EC cells were isolated by sorting for 2N DNA content or >4N DNA content using a BD FCASAria Fusion flow cytometer after incubation with Hoechst 33342 (5 µg/ml, Thermo) at 37 °C for 30 min, or DyeCycle Ruby (1:10,000, Thermo, V10309) for Fucci cells at 37 °C for 15 min.

### Antibodies

Immunoblotting was performed using the following antibodies diluted in TBS supplemented with 0.1% Tween 20 and 5% milk powder or 3% BSA: cyclin E1 (1:1000, Santa Cruz, sc-247), beta-actin (1:1000, Santa Cruz, sc-81178), p-p53 S15 (1:1000, Cell Signalling, 9284), p-CHK1 S345 (1:1000, Cell Signalling, 2348), p-RPA S4/S8 (1:5000, Bethyl Laboratories, A300-245), HA.11 (1:1000, BioLegend, 16B12), p53 (1:1000, Santa Cruz, sc-126), p21 (1:1000, Cell Signalling, 2947), HA (1:1000, Santa Cruz, sc-805), alpha-Tubulin (1:4000, Sigma, T5168), POT1 (1:1000, Novus Biologicals, NB500-176), FBXW7 (1:1000, Proteintech, 55290-1-AP), p-CDK1 T14/Y15 (in-house by Julian Gannon), Cdh1 (1:1000, in-house by Julian Gannon, AR38.2), anti-Mouse HRP (1:5000, Dako, P0447) and anti-Rabbit HRP (1:5000, Jackson Immuno, 711-035-152). The following antibodies were used for FACS and diluted in PBS supplemented with 1% BSA: MCM7 (1:200, Santa Cruz, sc-56324), cyclin B1 (1:200, Abcam, ab32053), p-H2A.X S139 (1:200, Millipore, 05-636), anti-Rabbit Alexa Fluor 555 (1:500, Thermo, A21428) and anti-Mouse Alexa Fluor 555 (1:500, Thermo, A21424).

## Quantification and Statistical Analysis

Graphpad Prism were used for all statistical analyses. Statistical methods are described in the figure legends as appropriate.

## Supplementary Material

Supplemental information can be found online at https://doi.org/10.1016/j.cell.2022.12.036.

Video S1

Video S2

Video S3

Video S4

Video S5

Video S6

Supplemental figures

## Data Availability

Original immunoblot images have been deposited at Figshare and are publicly available (DOI: https://doi.org/10.6084/m9.figshare.c.6284868.v1). FIJI macro for FUCCI analysis has been deposited at GitHub (https://github.com/zeng-j-k/Cell-FUCCI-analysis.git) and is publicly available as of the date of publication. Any additional information required to reanalyse the data reported in this paper is available from the lead contact upon request.

## Highlights

- Replicative stress from elevated cyclin E drives p53-dependent mitotic bypass
- p53, via p21, promotes mitotic bypass by inhibiting cyclin-dependent kinase
- Elevated cyclin E drives p53+/+ G1-arrested cells to complete endoreduplication
- Cyclin E can therefore drive WGD in p53-proficient cells

## In brief

DNA replication stress-induced whole-genome duplication (WGD) requires p53 function, in contrast to WGD induced by DNA damage; these findings provide mechanistic insight into cancer genome evolution in p53^+/+^ tumors.

## References

[1] Taylor AM, Shih J, Ha G, Gao GF, Zhang X, Berger AC, Schumacher SE, Wang C, Hu H, Liu J (2018). Genomic and functional approaches to understanding cancer aneuploidy. Cancer Cell.

[2] Davoli T, de Lange T (2011). The causes and consequences of polyploidy in normal development and cancer. Annu Rev Cell Dev Biol.

[3] Zack TI, Schumacher SE, Carter SL, Cherniack AD, Saksena G, Tabak B, Lawrence MS, Zhsng C-Z, Wala J, Mermel CH (2013). Pan-cancer patterns of somatic copy number alteration. Nat Genet.

[4] Bielski CM, Zehir A, Penson AV, Donoghue MTA, Chatila W, Armenia J, Chang MT, Schram AM, Jonsson P, Bandlamudi C (2018). Genome doubling shapes the evolution and prognosis of advanced cancers. Nat Genet.

[5] Quinton RJ, DiDomizio A, Vittoria MA, Kotýnková A, Ticas CJ, Patel S, Koga Y, Vakhshoorzadeh J, Hermance N, Kuroda TS (2021). Whole-genome doubling confers unique genetic vulnerabilities on tumour cells. Nature.

[6] Duelli DM, Padilla-Nash HM, Berman D, Murphy KM, Ried T, Lazebnik Y (2007). A virus causes cancer by inducing massive chromosomal instability through cell fusion. Curr Biol.

[7] Sotillo R, Hernando E, Díaz-Rodríguez E, Teruya-Feldstein J, Cordón-Cardo C, Lowe SW, Benezra R (2007). Mad2 overexpression promotes aneuploidy and tumorigenesis in mice. Cancer Cell.

[8] Lens SMA, Medema RH (2019). Cytokinesis defects and cancer. Nat Rev Cancer.

[9] Davoli T, Denchi EL, de Lange T (2010). Persistent telomere damage induces bypass of mitosis and tetraploidy. Cell.

[10] Ganem NJ, Pellman D (2007). Limiting the proliferation of polyploid cells. Cell.

[11] Krenning L, Feringa FM, Shaltiel IA, van den Berg J, Medema RH (2014). Transient activation of p53 in G2 phase is sufficient to induce senescence. Mol Cell.

[12] Johmura Y, Shimada M, Misaki T, Naiki-Ito A, Miyoshi H, Motoyama N, Ohtani N, Hara E, Nakamura M, Morita A (2014). Necessary and sufficient role for a mitosis skip in senescence induction. Mol Cell.

[13] Gorgoulis VG, Vassiliou LV, Karakaidos P, Zacharatos P, Kotsinas A, Liloglou T, Venere M, Ditullio RA, Kastrinakis NG, Levy B (2005). Activation of the DNA damage checkpoint and genomic instability in human precancerous lesions. Nature.

[14] Bartkova J, Horejsí Z, Koed K, Krämer A, Tort F, Zieger K, Guldberg P, Sehested M, Nesland JM, Lukas C (2005). DNA damage response as a candidate anti-cancer barrier in early human tumorigenesis. Nature.

[15] Bester AC, Roniger M, Oren YS, Im MM, Sarni D, Chaoat M, Bensimon A, Zamir G, Shewach DS, Kerem B (2011). Nucleotide deficiency promotes genomic instability in early stages of cancer development. Cell.

[16] Ekholm-Reed S, Méndez J, Tedesco D, Zetterberg A, Stillman B, Reed SI (2004). Deregulation of cyclin E in human cells interferes with prereplication complex assembly. J Cell Biol.

[17] Macheret M, Halazonetis TD (2018). Intragenic origins due to short G1 phases underlie oncogene-induced DNA replication stress. Nature.

[18] Matson JP, Dumitru R, Coryell P, Baxley RM, Chen W, Twaroski K, Webber BR, Tolar J, Bielinsky A-K, Purvis JE (2017). Rapid DNA replication origin licensing protects stem cell pluripotency. eLife.

[19] Yeh CH, Bellon M, Nicot C (2018). FBXW7: a critical tumor suppressor of human cancers. Mol Cancer.

[20] Sakaue-Sawano A, Kurokawa H, Morimura T, Hanyu A, Hama H, Osawa H, Kashiwagi S, Fukami K, Miyata T, Miyoshi H (2008). Visualizing spatiotemporal dynamics of multicellular cell-cycle progression. Cell.

[21] Sakaue-Sawano A, Yo M, Komatsu N, Hiratsuka T, Kogure T, Hoshida T, Goshima N, Matsuda M, Miyoshi H, Miyawaki A (2017). Genetically encoded tools for optical dissection of the mammalian cell cycle. Mol Cell.

[22] Clijsters L, Ogink J, Wolthuis R (2013). The spindle checkpoint, APC/C(Cdc20), and APC/C(Cdh1) play distinct roles in connecting mitosis to S phase. J Cell Biol.

[23] Jones RM, Mortusewicz O, Afzal I, Lorvellec M, García P, Helleday T, Petermann E (2013). Increased replication initiation and conflicts with transcription underlie cyclin E-induced replication stress. Oncogene.

[24] Neelsen KJ, Zanini IM, Herrador R, Lopes M (2013). Oncogenes induce genotoxic stress by mitotic processing of unusual replication intermediates. J Cell Biol.

[25] Tanaka S, Diffley JF (2002). Deregulated G1-cyclin expression induces genomic instability by preventing efficient pre-RC formation. Genes Dev.

[26] Ganem NJ, Godinho SA, Pellman D (2009). A mechanism linking extra centrosomes to chromosomal instability. Nature.

[27] Chen S, Stout JR, Dharmaiah S, Yde S, Calvi BR, Walczak CE (2016). Transient endoreplication down-regulates the kinesin-14 HSET and contributes to genomic instability. Mol Biol Cell.

[28] Gemble S, Wardenaar R, Keuper K, Srivastava N, Nano M, Macé AS, Tijhuis AE, Bernhard SV, Spierings DCJ, Simon A (2022). Genetic instability from a single S phase after whole-genome duplication. Nature.

[29] Irani K, Xia Y, Zweier JL, Sollott SJ, Der CJ, Fearon ER, Sundaresan M, Finkel T, Goldschmidt-Clermont PJ (1997). Mitogenic signaling mediated by oxidants in Ras-transformed fibroblasts. Science.

[30] Lee AC, Fenster BE, Ito H, Takeda K, Bae NS, Hirai T, Yu ZX, Ferrans VJ, Howard BH, Finkel T (1999). Ras proteins induce senescence by altering the intracellular levels of reactive oxygen species. J Biol Chem.

[31] Park YB, Park MJ, Kimura K, Shimizu K, Lee SH, Yokota J (2002). Alterations in the INK4a/ARF locus and their effects on the growth of human osteosarcoma cell lines. Cancer Genet Cytogenet.

[32] Sherr CJ (2001). The INK4a/ARF network in tumour suppression. Nat Rev Mol Cell Biol.

[33] Bunz F, Dutriaux A, Lengauer C, Waldman T, Zhou S, Brown JP, Sedivy JM, Kinzler KW, Vogelstein B (1998). Requirement for p53 and p21 to sustain G2 arrest after DNA damage. Science.

[34] Taylor WR, Stark GR (2001). Regulation of the G2/M transition by p53. Oncogene.

[35] Rata S, Suarez Peredo Rodriguez MF, Joseph S, Peter N, Echegaray Iturra F, Yang F, Madzvamuse A, Ruppert JG, Samejima K, Platani M (2018). Two interlinked bistable switches govern mitotic control in mammalian cells. Curr Biol.

[36] Zielke N, Edgar BA, DePamphilis ML (2013). Endoreplication. Cold Spring Harb Perspect Biol.

[37] Mailand N, Diffley JF (2005). CDKs promote DNA replication origin licensing in human cells by protecting Cdc6 from APC/C-dependent proteolysis. Cell.

[38] Lukas C, Sørensen CS, Kramer E, Santoni-Rugiu E, Lindeneg C, Peters JM, Bartek J, Lukas J (1999). Accumulation of cyclin B1 requires E2F and cyclin-A-dependent rearrangement of the anaphase-promoting complex. Nature.

[39] Soussi T, Wiman KG (2015). TP53: an oncogene in disguise. Cell Death Differ.

[40] Ohrt T, Merkle D, Birkenfeld K, Echeverri CJ, Schwille P (2006). In situ fluorescence analysis demonstrates active siRNA exclusion from the nucleus by Exportin 5. Nucleic Acids Res.

[41] Tinevez JY, Perry N, Schindelin J, Hoopes GM, Reynolds GD, Laplantine E, Bednarek SY, Shorte SL, Eliceiri KW (2017). TrackMate: an open and extensible platform for single-particle tracking. Methods.

[42] Rodríguez-Martínez M, Hills SA, Diffley JFX, Svejstrup JQ (2020). Multiplex cell fate tracking by flow cytometry. Methods Protoc.

