# Supplementary material for: Cyclin E-induced replicative stress drives p53-dependent whole-genome duplication
Source: Cell. Author manuscript; Available in PMC 2026 Aug 26. (PMC7619399; doi:10.1016/j.cell.2022.12.036)
Supplement: Supplementary file 1 [file EMS217831-supplement-Supplemental_figures.pdf]

Supplemental figures

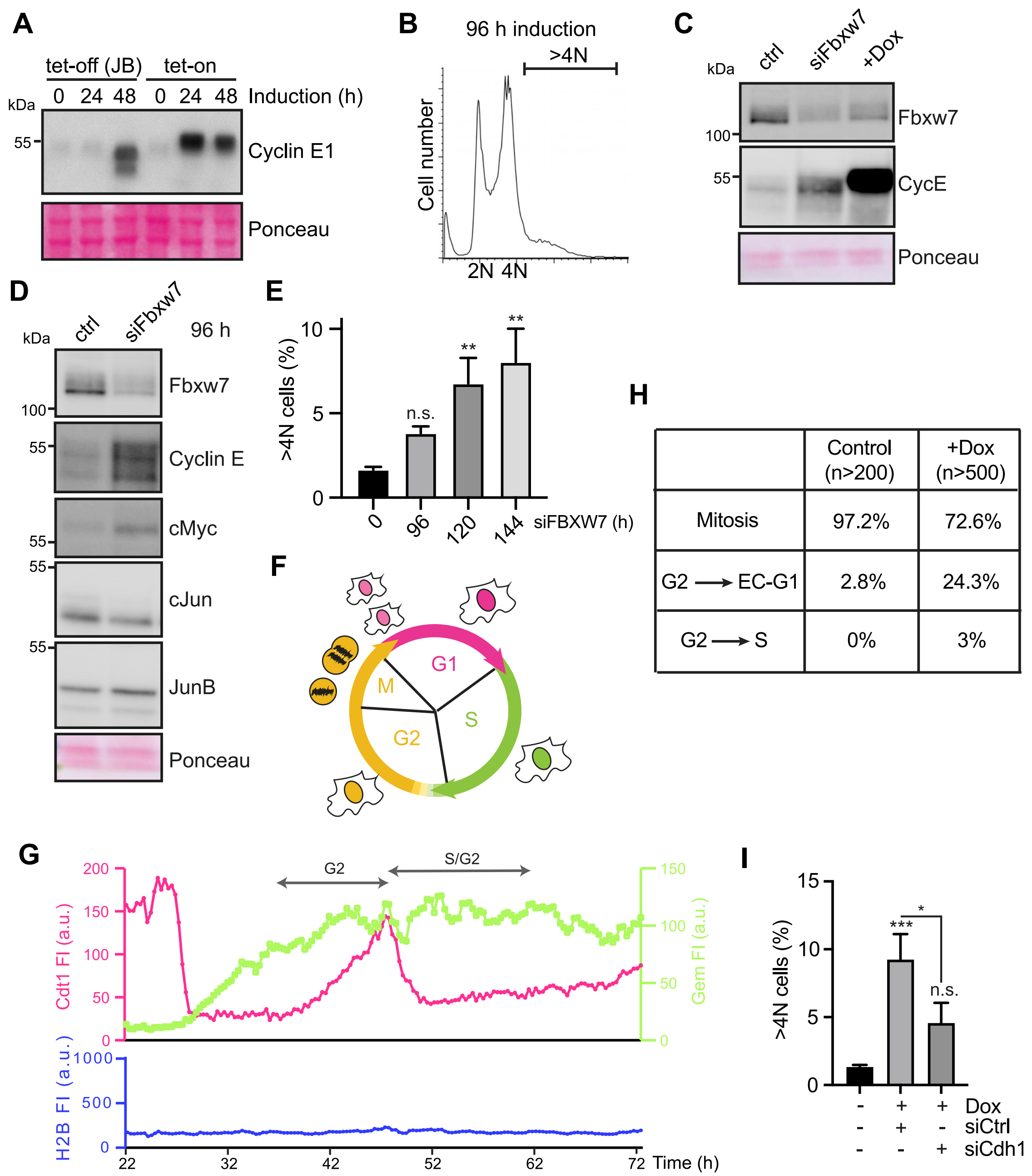

	Control (n>200)	+Dox (n>500)
Mitosis	97.2%	72.6%
G2 ⟶ EC-G1	2.8%	24.3%
G2 ⟶ S	0%	3%

Figure S1. Aberrant G2 transitions in cyclin E-expressing cells; involvement of Cdh1 in CycE-induced endoreduplication, related to Figure 1

(A) Immunoblots showing cyclin E1 induction in U2OS tetON CycE cell line and U2OS tetOFF CycE cell line.

(B) FACS DNA content analysis of U2OS cells expressing cyclin E.

(C and D) Immunoblots showing expression of oncogenes after knockdown of Fbxw7 in U2OS cells.

(E) U2OS cells depleted with Fbxw7 were analyzed by FACS over time. Quantification of >4N cells with SDs is shown (n = 3). Statistical significance: **$p < 0.005$, Tukey's method.

(F) Schematic showing colors of cell-cycle phases of the FUCCI system.

(G) Temporal profiles of a cyclin E-expressing U2OS cell that degraded mCherry-Cdt1 while maintaining high level of mVenus-Gem.

(H) The percentages of cell-cycle transitions from G2 in control and cyclin E-expressing (+ Dox) U2OS cells.

(I) U2OS cells were induced to express cyclin E by Dox for 96 h and were treated with siRNAs at 48 h to knock down Cdh1. Quantification of the percentage of >4N cells by FACS analysis with SDs is shown (n = 3). Statistical significance: *$p < 0.05$; ***$p < 0.001$, Tukey's method.

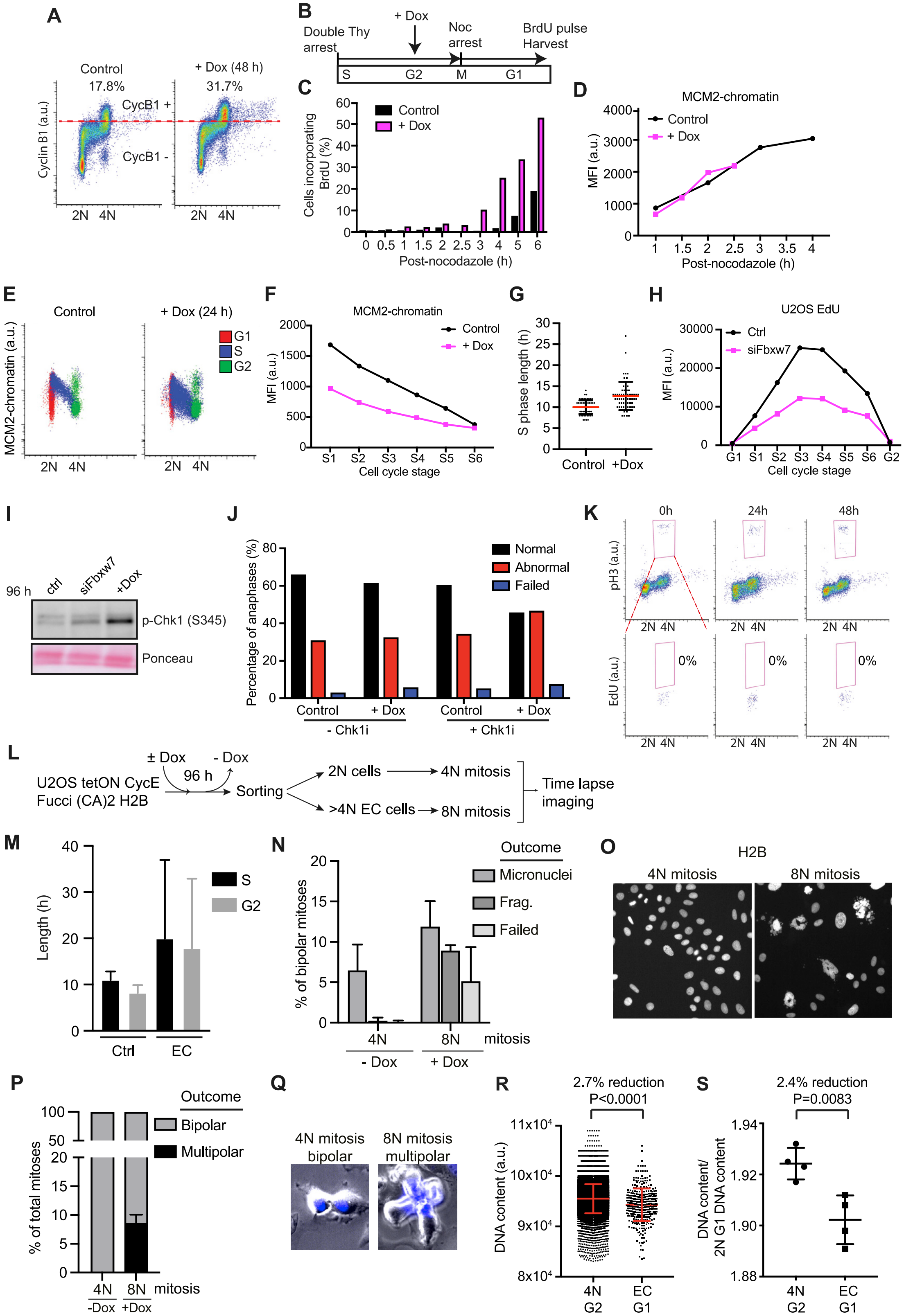

(legend on next page)

CellPress
OPEN ACCESS

Figure S2. Characterization of replicative stress in cyclin E-expressing cells and endoreduplicated cells, related to Figure 2

(A) FACS analysis of cyclin E-expressing U2OS cells for cyclin B1 level and DNA content.

(B–D) U2OS cells were synchronized using double thymidine and nocodazole, and treated as in (B). Quantification of the percentage of cells incorporating BrdU by FACS at different time points is shown in (C). The level of chromatin-bound MCM2 is shown in (D).

(E) FACS analysis of chromatin bound MCM2 level on cyclin E-expressing U2OS cells. G1, S, and G2 phases were colored; G1: 2N, EdU-. S: 2N-4N, EdU+. G2: 4N, EdU-.

(F) S phase was divided as in Figure 2D. Chromatin-bound MCM2 level was analyzed by FACS in cyclin E-expressing U2OS cells.

(G) S phase lengths of cells measured in FUCCI live-cell imaging. At least 60 cells were analyzed for each condition.

(H) EdU incorporation analysis by FACS in siFbxw7-treated cells.

(I) Immunoblot showing phosphorylated Chk1 (S345) expression in siFbxw7-treated cells.

(J) U2OS H2B-mCherry cells were induced to express cyclin E for 24 h and then treated with 100 nM Chk1 inhibitor and live cell imaged for 12 h. Quantification of abnormal and failed mitosis is shown.

(K) U2OS cells expressing cyclin E were stained with pH3 and EdU. Mitotic cells were identified as pH3+ cells. The percentage of EdU+ cells is shown.

(L–Q) Schematic of the experimental approach (L). Mean lengths ± SDs of S and G2 phases of sorted 2N and >4N EC cells are shown in (M). The percentage of bipolar mitoses ending with micronucleus generation, nuclear fragmentation (Frag.), or cytokinesis failure (Failed) are shown in (N). Example still images of the H2B-mTurquiose channel are shown in (O). The percentages of bipolar and multipolar mitosis are shown in (P). Example composite images of H2B-mTurquoise (blue) and phase-contrast channels for bipolar and multipolar mitosis are shown in (Q).

(R and S) Cells in Figure 2H were stained with DAPI for DNA content analysis. DNA content level for individual cells in a representative experiment is shown in (R) and relative DNA content is averaged as shown in (S) (n = 4). Statistical significance was examined by unpaired t test.

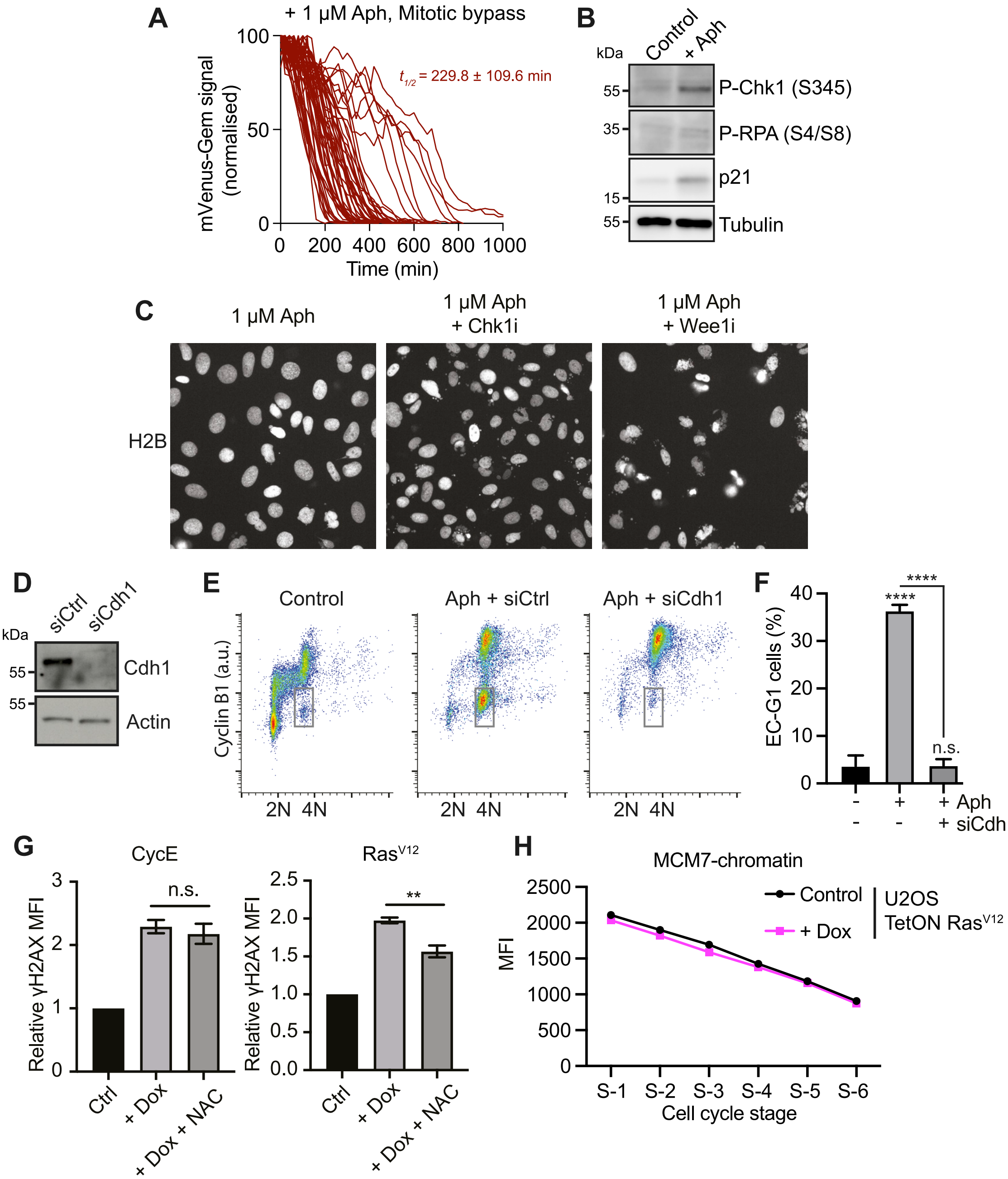

Figure S3. Involvement of the G2 checkpoint and Cdh1 in aphidicolin-induced mitotic bypass; replicative stress in Ras^{V12}-expressing cells, related to Figure 3

(A) Normalized data on the time-course degradation of mVenus-Gem. Each line represents a single-cell tracking (n > 50). Mean half-life ($t_{1/2}$) values with SDs of mVenus-Gem degradation are shown in the figure and main text.

(B) Immunoblots showing DNA damage markers after 72-h 1 μM aphidicolin treatment in U2OS cells.

(C) Example still images of the H2B-mTurquiose channel of cells in Figure 3B.

(D–F) U2OS cells treated with 1 μM Aph were incubated with siRNA to knockdown Cdh1. Immunoblots showing Cdh1 knockdown are in (D). Cells were analyzed by FACS (E). The mean percentage of EC-G1 cells with SDs is shown in (F) (n = 3). Statistical significance: ****$p < 0.0001$, Tukey's method.

(G) U2OS cells induced (+ Dox) to express CycE or Ras^{V12} were incubated with 5mM N-acetyl cysteine (NAC). Quantification of >4N cells by FACS analysis with SDs is shown (n = 3). Statistical significance: **$p < 0.01$, paired t test.

(H) Mean fluorescence intensities (MFI) for chromatin-bound MCM of Ras^{V12}-expressing U2OS cells.

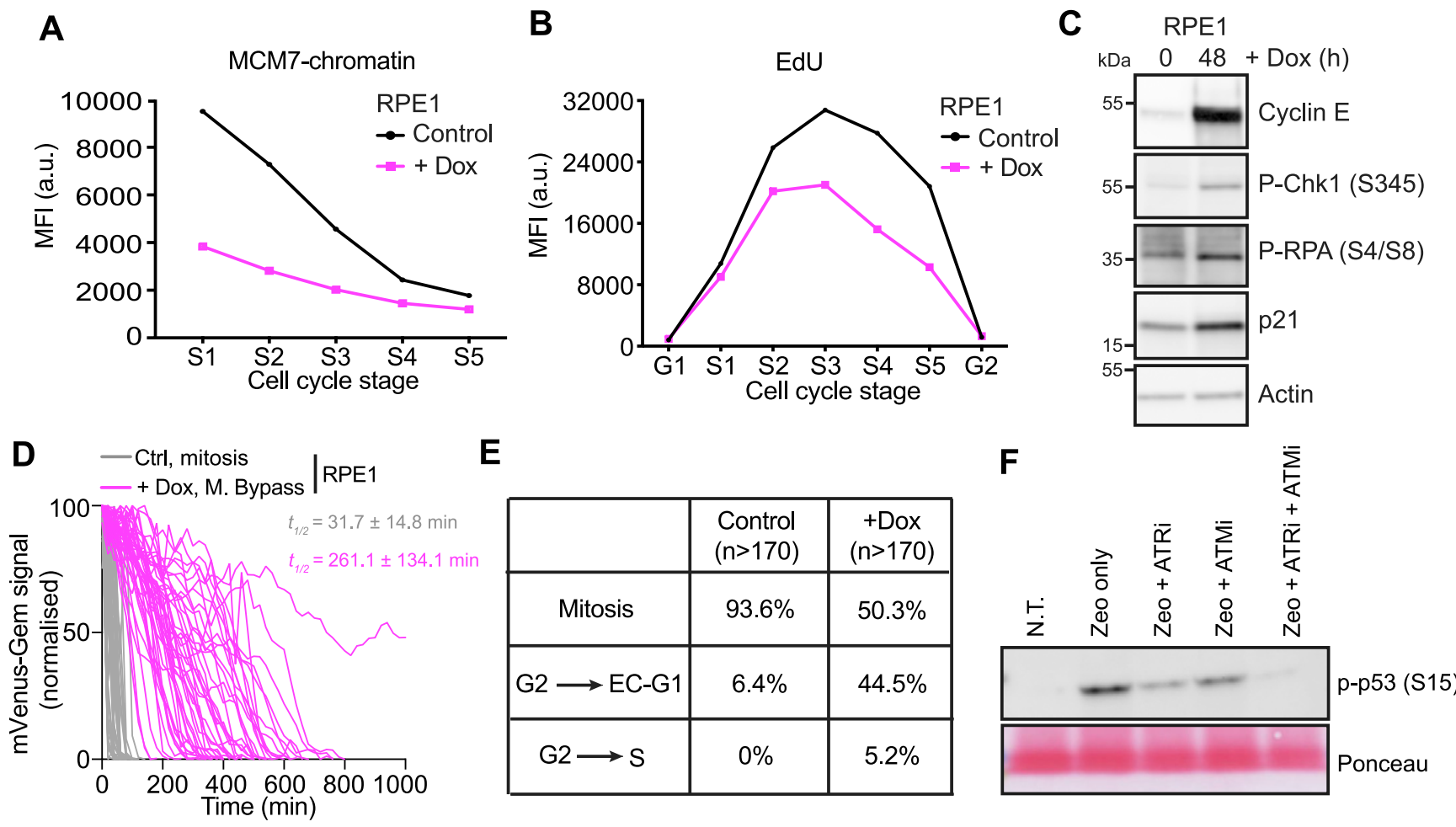

	Control (n>170)	+Dox (n>170)
Mitosis	93.6%	50.3%
G2 → EC-G1	6.4%	44.5%
G2 → S	0%	5.2%

Figure S4. Characterization of replicative stress in cyclin E-expressing RPE1 cells; aberrant G2 transitions, related to Figure 4

(A and B) RPE1 cells were analyzed by FACS as in Figure 2D.

(C) Immunoblots showing DNA damage markers.

(D) Normalized time-course degradation of mVenus-Gem in cyclin E-expressing RPE1 cells that bypassed mitosis (n > 40 for each condition). Mean half-life ($t_{1/2}$) values with SDs are shown in the figure and main text.

(E) The percentages of cell-cycle transitions from G2 in cyclin E-expressing RPE1 cells.

(F) To show the ATM and ATR inhibitors are functional, we added the inhibitors to zeocin-treated U2OS cells that are supposed to activate both ATM and ATR pathways. Immunoblot showing phosphorylated p53 (S15) expression in zeocin-treated U2OS cells after 6-h supplementation with inhibitors of ATR or ATM.

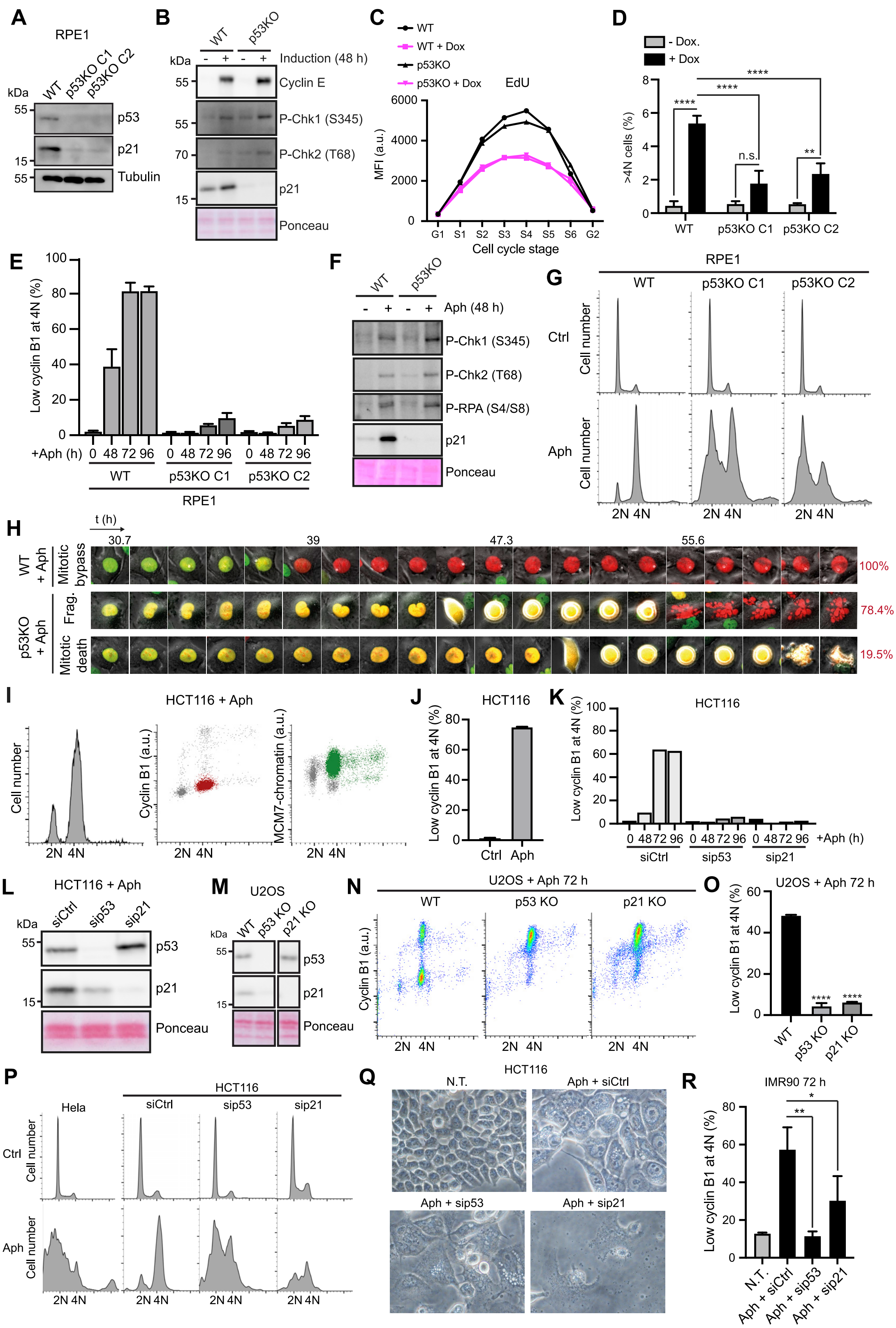

(legend on next page)

Figure S5. Involvement of p53 and p21 in aphidicolin-induced mitotic bypass, related to Figure 5

(A) Immunoblots showing knockout of p53 in RPE1 cells (clone #1&2).
(B) Immunoblots showing DNA damage markers in RPE1 WT and p53 KO cells expressing cyclin E.
(C) EdU incorporation analysis by FACS of RPE1 WT and p53 KO cells expressing cyclin E.
(D) Quantification of RPE1 WT and p53 KO cells with >4N DNA content after 96-h expression of cyclin E. Mean percentages with SDs are shown (n = 3). Statistical significance: **$p < 0.005$; ****$p < 0.0001$, Tukey's method.
(E) Cells were treated with 1 μM aphidicolin and analyzed for cyclin B level and DNA content by FACS. The mean percentage of EC-G1 cells with range is shown (n = 2).
(F) Immunoblots showing DNA damage markers.
(G) DNA content analysis by FACS on cells treated with 1 μM aphidicolin for 72 h.
(H) Images of example cells in Figure 5D showing mitotic bypass, mitotic death, and nuclear fragmentation are shown. The mean percentage of different outcomes is shown on the right (n = 3).
(I) HCT116 cells were treated with 1 μM aphidicolin for 72 h before being analyzed for cyclin B level, chromatin-bound MCM7 level, and DNA content by FACS. EC-G1 cells are identified and labeled in red. Cells with high MCM7 at 4N or >4N DNA content are labeled in green.
(J) Quantification of the percentage of EC-G1 cells in (I) with range is shown (n = 2).
(K) HCT116 cells were treated with 1 μM aphidicolin, supplemented with siRNAs, and analyzed as in (E).
(L) Immunoblots showing knockdown of p53 and p21 in HCT116 cells.
(M) Immunoblots showing knockout of p53 and p21 in U2OS cells.
(N and O) Cells were treated with 1 μM aphidicolin for 72 h before being analyzed for cyclin B by FACS (N). The percentage of EC-G1 cells with range is shown in (O) (n = 2). Statistical significance: ****$p < 0.0001$, Tukey's method.
(P) Hela and HCT116 cells were treated with 1 μM Aph and siRNAs were used to knock down p53 and p21 in HCT116 cells for 72 h. DNA content analysis by FACS is shown.
(Q) Representative wide-field images of HCT116 in (P).
(R) IMR90 cells were treated as in (P). Quantification of the percentage of EC-G1 cells with SDs is shown (n = 3). Statistical significance: *$p < 0.05$ and **$p < 0.005$, Tukey's method.

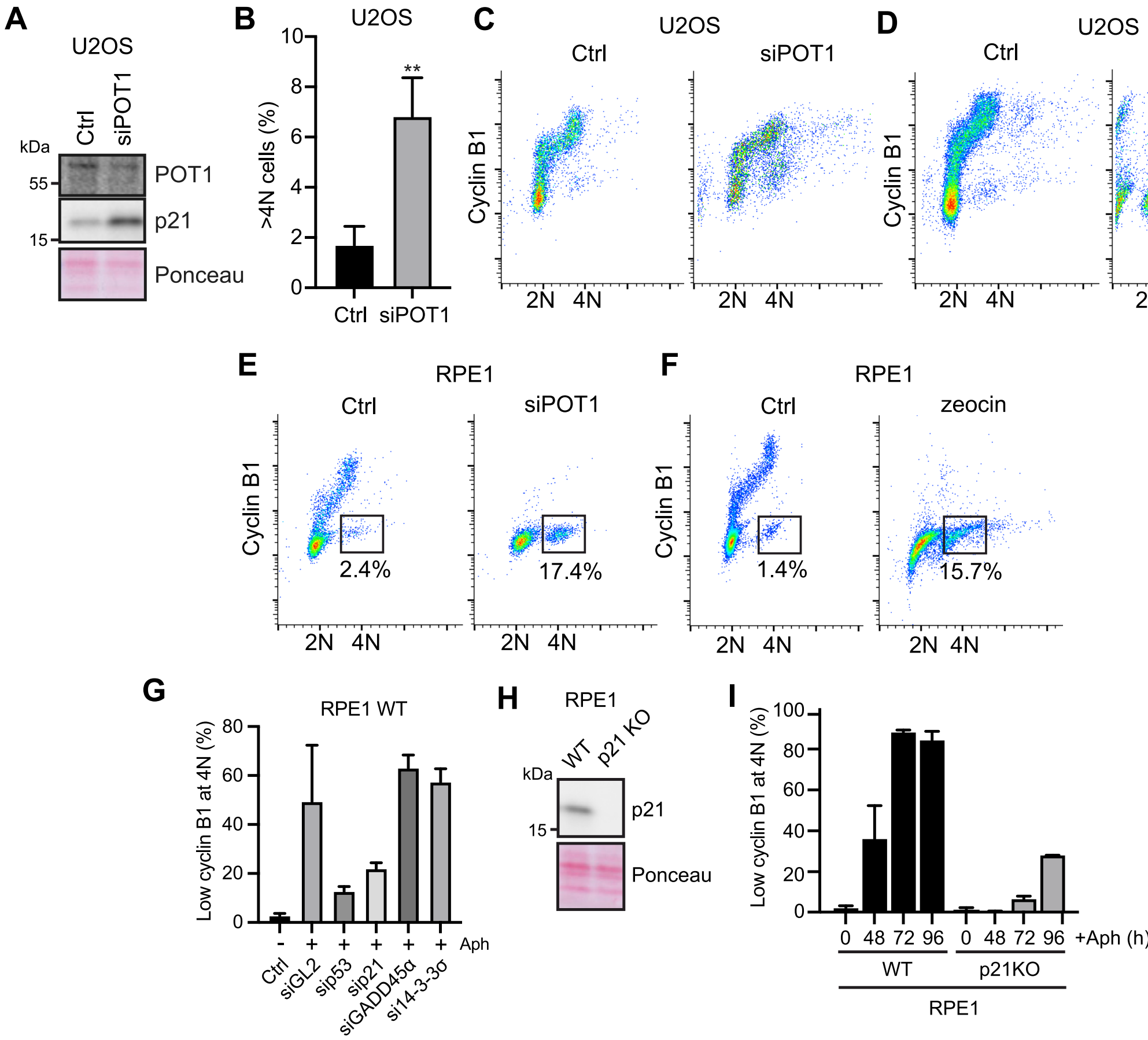

Figure S6. POT1 knockdown-induced mitotic bypass, involvement of p21 in mitotic bypass, related to Figure 5

(A) Immunoblots showing POT1 and p21 in POT1-depleted U2OS cells.

(B) POT1-depleted U2OS cells were analyzed by FACS. Quantification of >4N cells with SDs is shown (n = 3). Statistical significance: $^{**}p < 0.005$, unpaired t test.

(C) FACS analysis of U2OS cells treated with siPOT1 for 96 h.

(D) FACS analysis of U2OS cells treated with 100 μg/ml zeocin for 96 h.

(E) FACS analysis of depleted with POT1 for 96 h. Numbers indicate the percentage of EC-G1 cells.

(F) FACS analysis of cyclin B level and DNA content on RPE1 cells treated with 100 μg/ml zeocin for 96 h. Numbers indicate the percentage of cells with a low cyclin B level at 4N DNA content (EC-G1 cells).

(G) Downstream effectors of p53 were depleted in RPE1 WT cells treated with 0.5 μM aphidicolin for 72 h. Quantification of EG-G1 cells with SDs is shown (n = 3).

(H) Immunoblot showing knockout of p21 in RPE1 cells.

(I) RPE1 p21 KO cells were analyzed as in Figure S5E. The percentage of EC-G1 cells with range is shown (n = 2).

CellPress
OPEN ACCESS

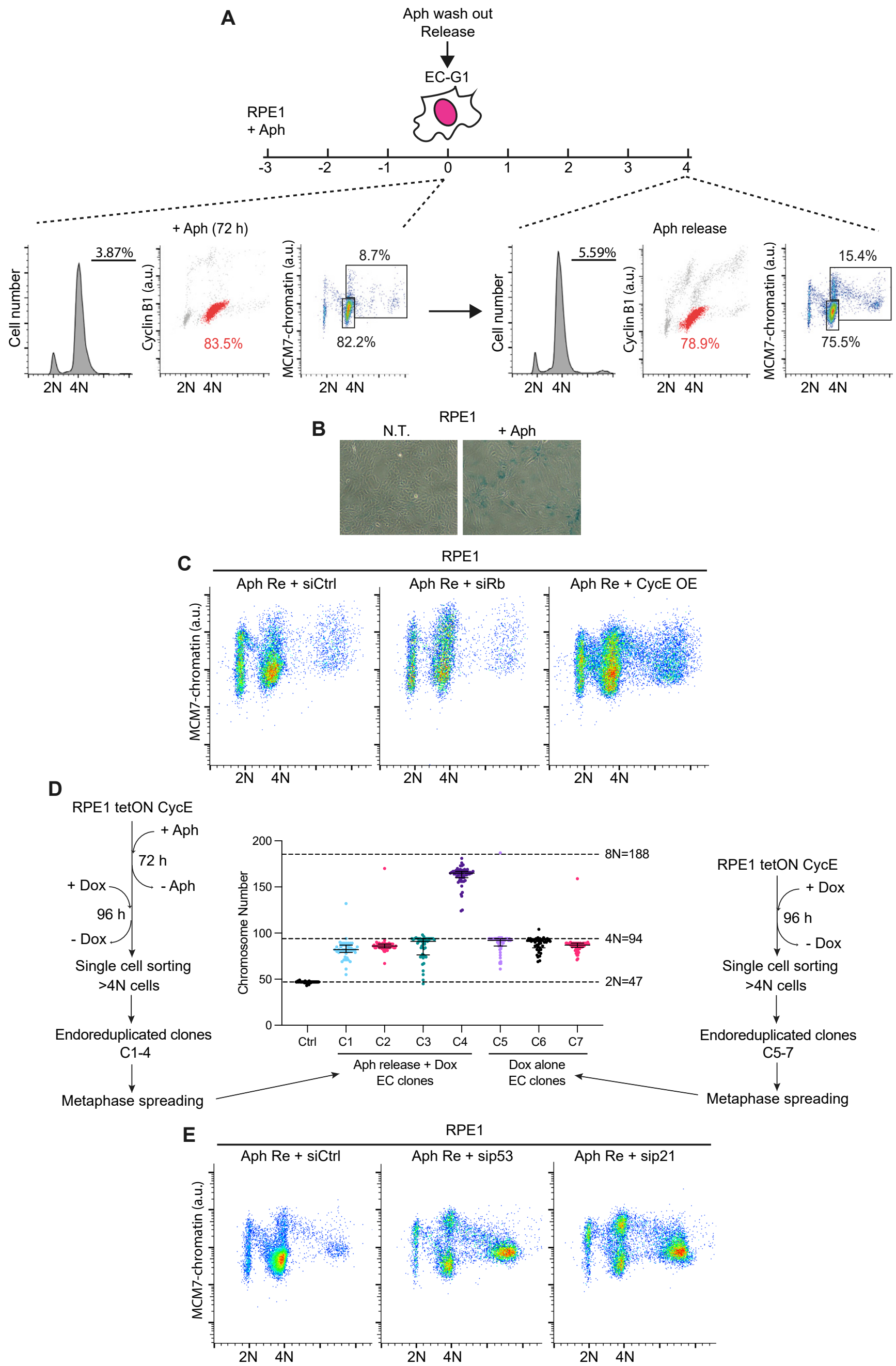

Figure S7. Knockdown of p53, p21, and Rb promotes cell-cycle re-entry of senescent EC-G1 cells, related to Figure 6

(A) Schematic of the experimental approach is shown on top. Cells were analyzed by FACS. EC-G1 cells are identified and labeled in red.

(B) Representative wide-field images of RPE1 cells treated with 1 μM aphidicolin for 96 h.

The blue color indicates β-galactosidase activity.

(C) RPE1 cells were treated as in Figure 6B and analyzed by FACS at 96 h post aphidicolin release for DNA content and MCM loading. siRNAs were used to knock down Rb at the point of release.

(D) Chromosome number analysis of RPE1 endoreduplicated single-cell clones.

(E) RPE1 cells were treated as in Figure 6B and analyzed by FACS at 96 h post aphidicolin release for DNA content and MCM loading. siRNAs were used to knock down p53 or p21 at the point of release.
